# Correction: Does chubby Can get lower grades than skinny Sophie? Using an intersectional approach to uncover grading bias in German secondary schools

**DOI:** 10.1371/journal.pone.0320170

**Published:** 2025-03-05

**Authors:** The PLOS One Staff

## Notice of Republication

The proper name “Can” is incorrectly capitalised in the article title. The correct title is: Does chubby Can get lower grades than skinny Sophie? Using an intersectional approach to uncover grading bias in German secondary schools. The correct citation is: Nennstiel R, Gilgen S (2024) Does chubby Can get lower grades than skinny Sophie? Using an intersectional approach to uncover grading bias in German secondary schools. PLoS ONE 19(7): e0305703. https://doi.org/10.1371/journal.pone.0305703

The publisher apologizes for the error.

## Supporting information

S1 File Originally published, uncorrected article. (PDF)

S2 File Republished, corrected article. (PDF)

## References

[1] Nennstiel R, Gilgen S. Does chubby Can get lower grades than skinny Sophie? Using an intersectional approach to uncover grading bias in German secondary schools. PLoS One. 2024;19(7):e0305703. doi: 10.1371/journal.pone.0305703 38959194 PMC11221685

