# Supplementary material for: Does chubby Can get lower grades than skinny Sophie? Using an intersectional approach to uncover grading bias in German secondary schools
Source: PLoS One. 2024 Jul 3;19(7):e0305703. doi: 10.1371/journal.pone.0305703 (PMC11221685; doi:10.1371/journal.pone.0305703)
Supplement: S1 Fig — (PDF) [file pone.0305703.s001.pdf]

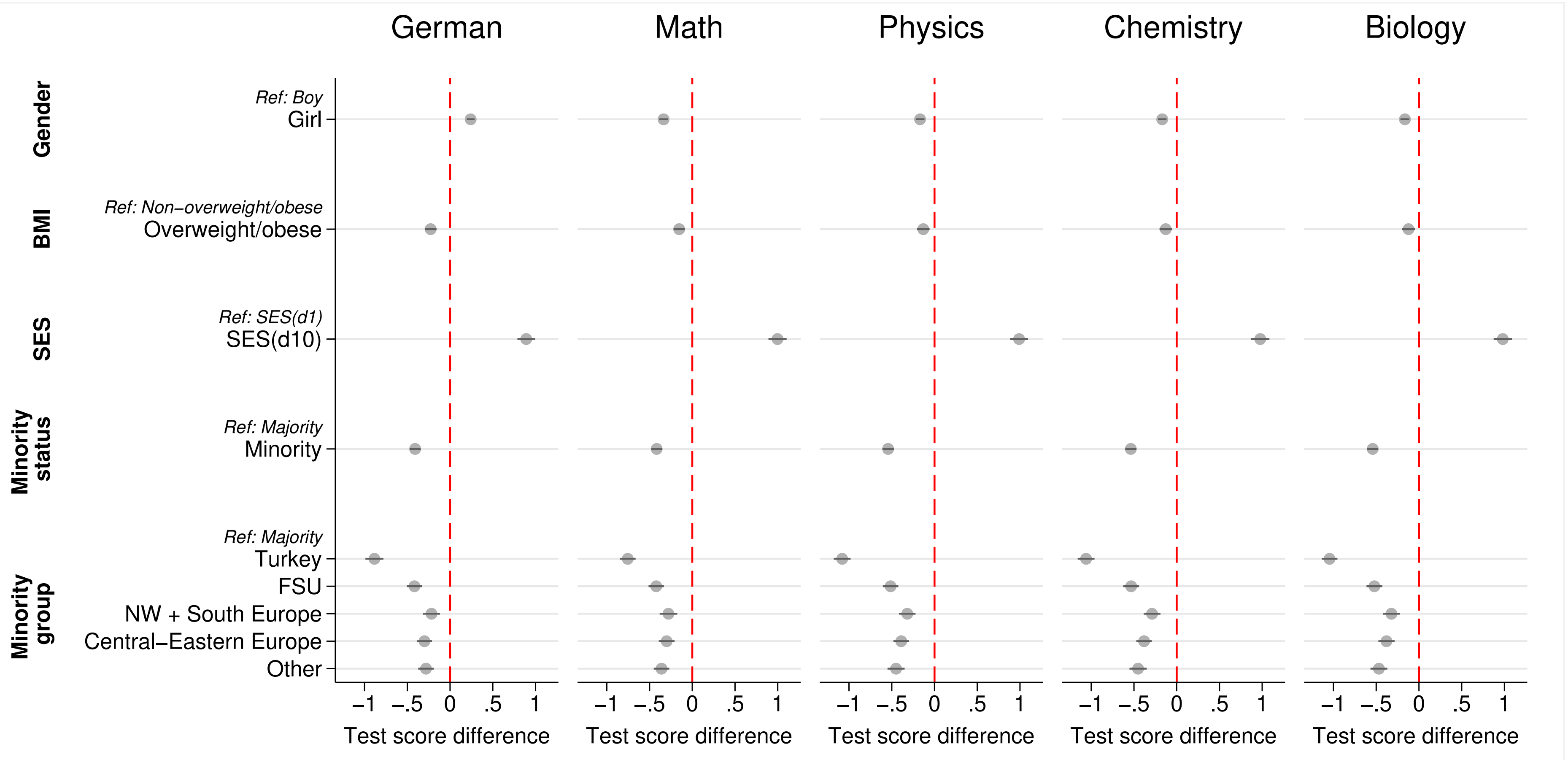

Figure S1: **Mean domain specific test score group differences across subjects.** Source: NEPS SC4 (based on m =50 multiple imputed datasets); weighted data, our own calculations.
