# Supplementary material for: Does chubby Can get lower grades than skinny Sophie? Using an intersectional approach to uncover grading bias in German secondary schools
Source: PLoS One. 2024 Jul 3;19(7):e0305703. doi: 10.1371/journal.pone.0305703 (PMC11221685; doi:10.1371/journal.pone.0305703)
Supplement: S2 Fig — Comparing majority to minority students. (PDF) [file pone.0305703.s002.pdf]

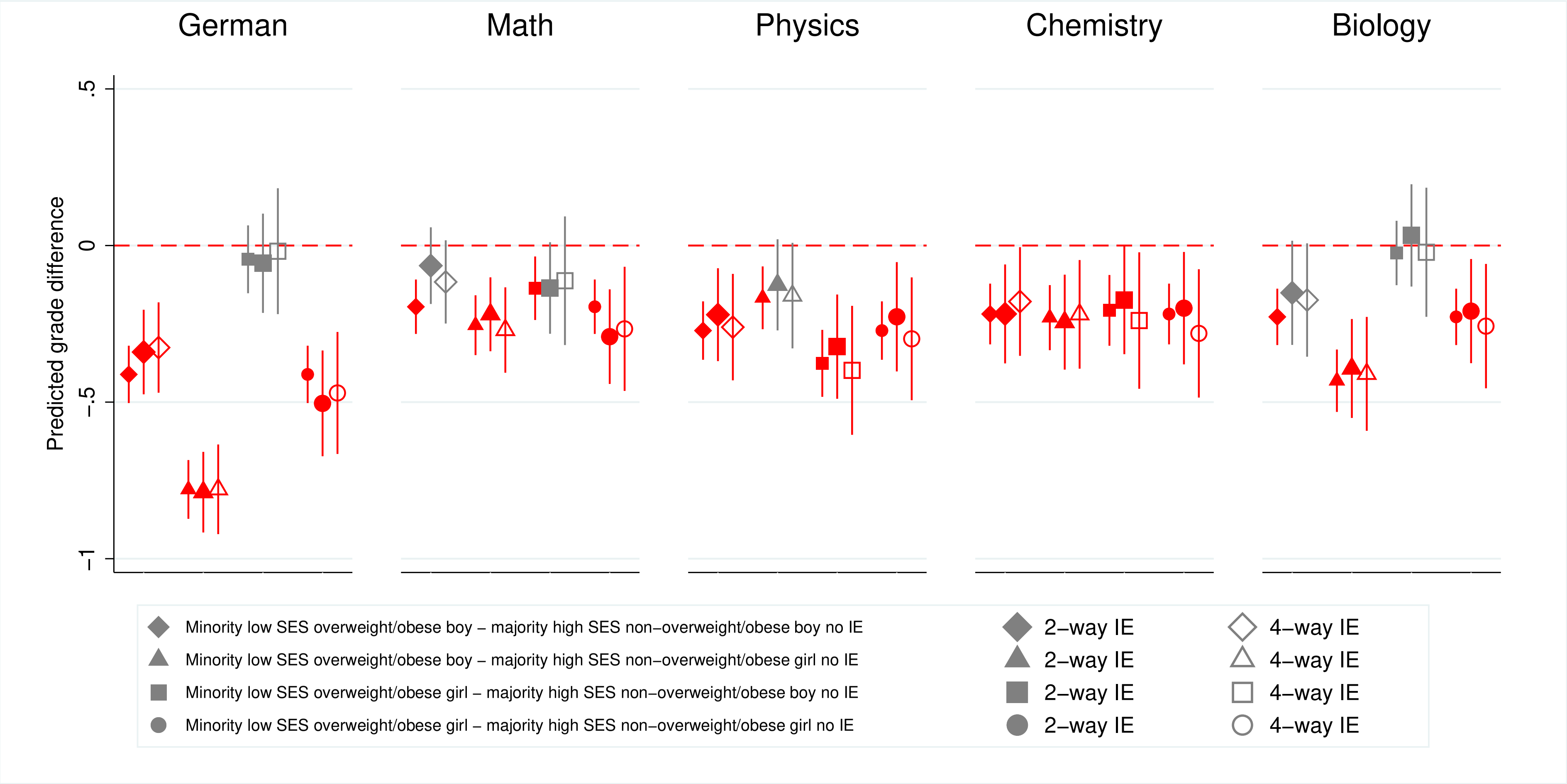

Figure S2: **Predicted school grade differences for students with specific ascriptive characteristics across subjects and different models. Comparing majority to minority students.** Note: Red colored icons indicate statistical significance. Predictive margins at the means of all other model variables. Predictions derived from multilevel regression models (see Tables S7-S11). No IE = models without interactions, 2-way IE = models with all 2-way interactions, 4-way IE = models with all 4-way interactions. Source: NEPS SC4 (based on m =50 multiple imputed datasets); weighted data, our own calculations.
