# Supplementary material for: Does chubby Can get lower grades than skinny Sophie? Using an intersectional approach to uncover grading bias in German secondary schools
Source: PLoS One. 2024 Jul 3;19(7):e0305703. doi: 10.1371/journal.pone.0305703 (PMC11221685; doi:10.1371/journal.pone.0305703)
Supplement: S3 Fig — (PDF) [file pone.0305703.s003.pdf]

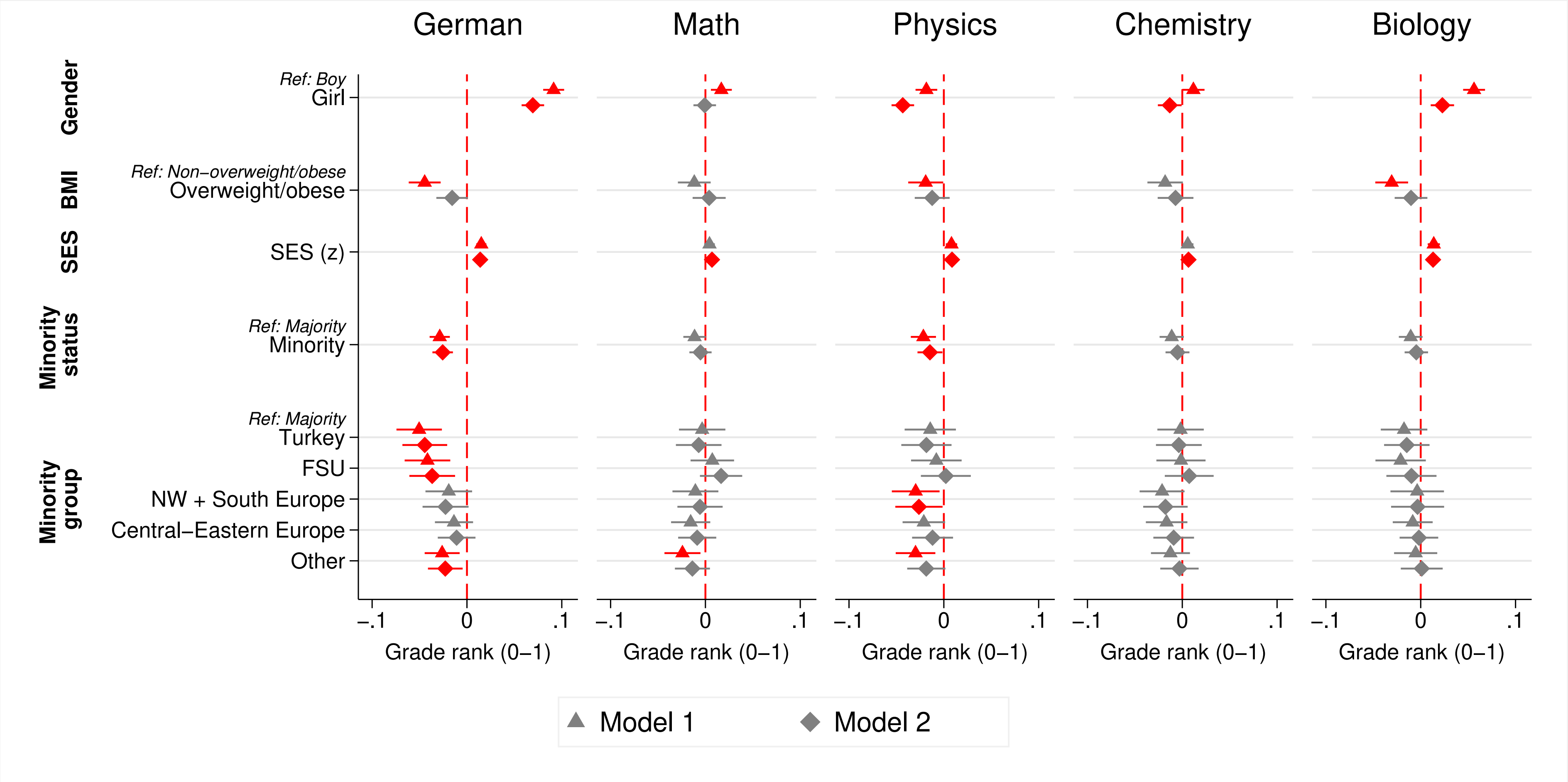

Figure S3: Student rank grading bias by students' ascriptive characteristics.
Note: Red colored icons indicate statistical significance. Regression coefficients based on linear regression models with clustered standard errors on school class level. Model 1 adjusted for domain specific competence, general academic competence and school track. Model 2 adjusted for all model variables).
Source: NEPS SC4 (based on m = 50 multiple imputed datasets); weighted data, our own calculations.
