# Supplementary material for: Does chubby Can get lower grades than skinny Sophie? Using an intersectional approach to uncover grading bias in German secondary schools
Source: PLoS One. 2024 Jul 3;19(7):e0305703. doi: 10.1371/journal.pone.0305703 (PMC11221685; doi:10.1371/journal.pone.0305703)
Supplement: S4 Fig — (PDF) [file pone.0305703.s004.pdf]

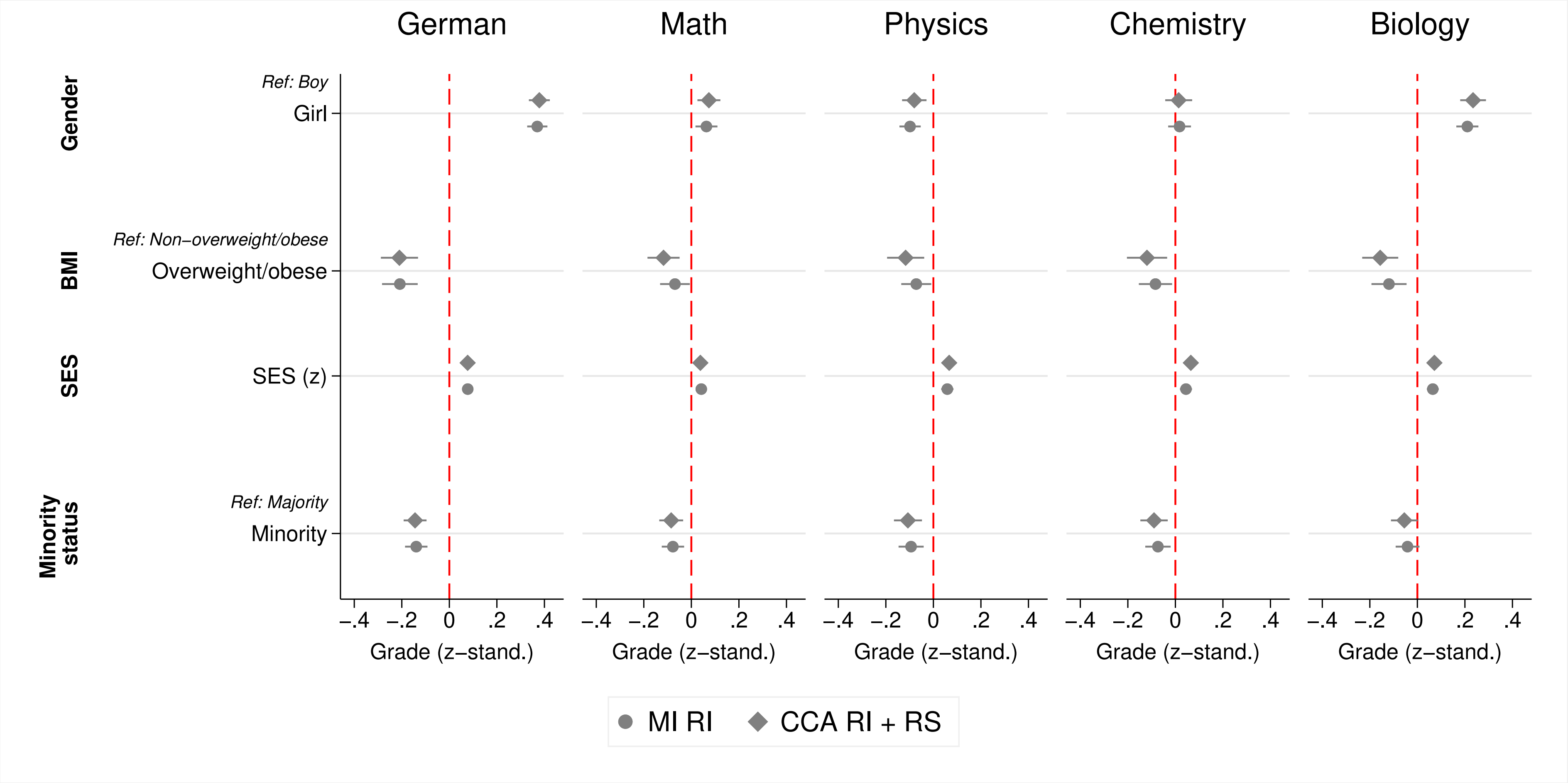

Figure S4: Effect comparison (model 1) of the random-intercept and random-intercept random-slope models.
Note: MI = multiple imputed data. RI = random-intercept model. CCA = complete case analysis (list-wise deleted data), RI + RS = random-intercept random-slope model. Regression coefficients based on three-level linear regression models. Model 1 adjusted for domain specific competence, general academic competence and school track.
Source: NEPS SC4 (based on m = 50 multiple imputed datasets); weighted data, our own calculations.
