# Supplementary material for: Does chubby Can get lower grades than skinny Sophie? Using an intersectional approach to uncover grading bias in German secondary schools
Source: PLoS One. 2024 Jul 3;19(7):e0305703. doi: 10.1371/journal.pone.0305703 (PMC11221685; doi:10.1371/journal.pone.0305703)
Supplement: S6 Fig — (PDF) [file pone.0305703.s006.pdf]

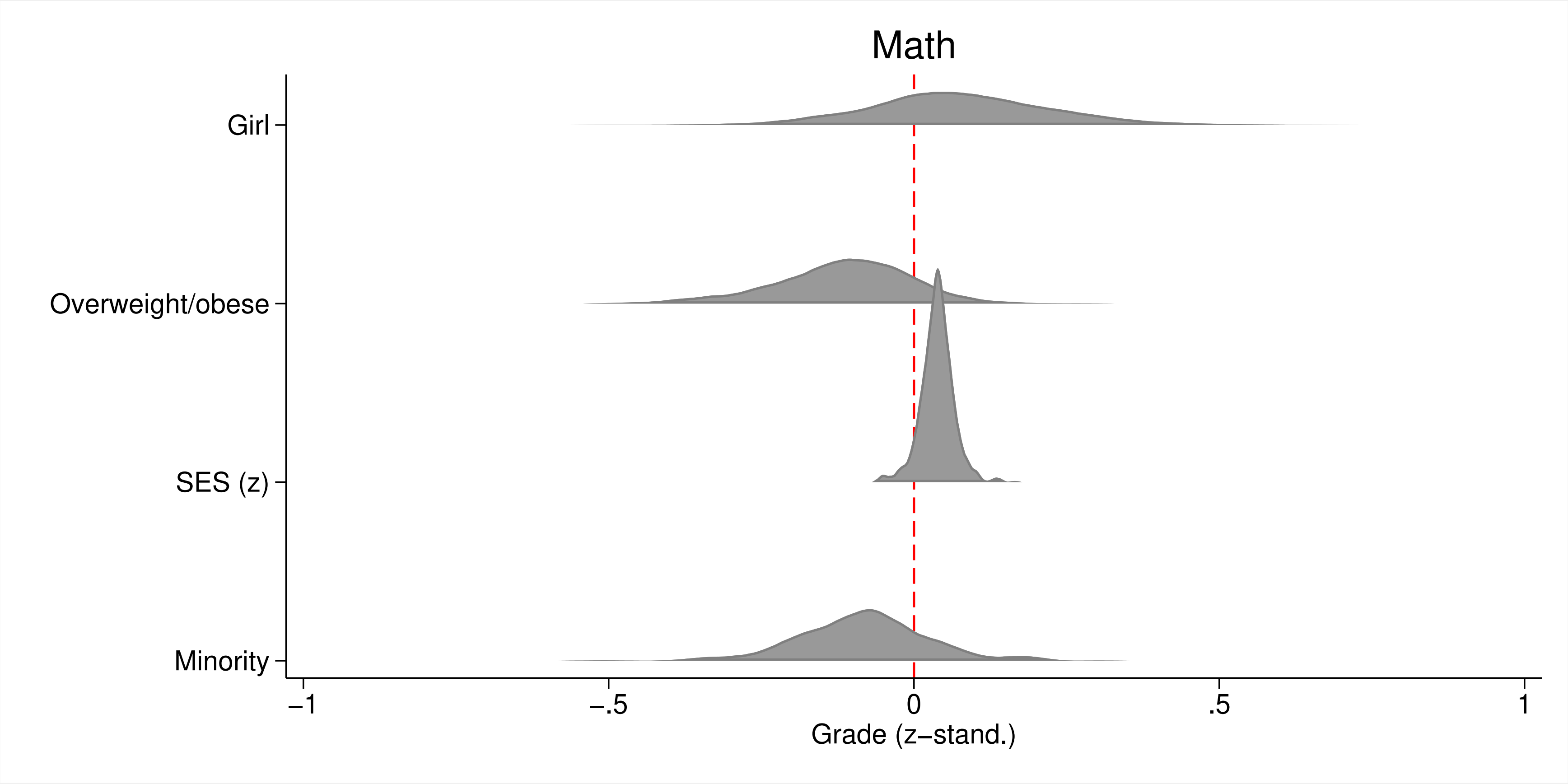

Figure S6: Distribution of the effects slopes of the ascriptive characteristics (model 1) on math grade.
Note: Estimates based on random-intercept random-slope three-level linear regression models run on list-wise deleted data (see Figure S2). Each school class represents one case. Only school classes with at least 10 students and at least one student with and one student without the ascriptive characteristic under study are taken into account.
Source: NEPS SC4 (based on m = 50 multiple imputed datasets); weighted data, our own calculations.
