# Supplementary material for: Does chubby Can get lower grades than skinny Sophie? Using an intersectional approach to uncover grading bias in German secondary schools
Source: PLoS One. 2024 Jul 3;19(7):e0305703. doi: 10.1371/journal.pone.0305703 (PMC11221685; doi:10.1371/journal.pone.0305703)
Supplement: S1 Table — (PDF) [file pone.0305703.s010.pdf]

Table S1: Information on potential sample sizes, sample sizes and missing value patterns.

Grade first survey wave		9
Potential sample size first survey wave		15,239
Students participating in first survey wave		15,017
Students participating in survey wave under investigation (sample size)		14,090
Proportion of sample size on potential sample size first survey wave		92.5
Proportion of sample size on students participating in first survey wave		93.8
**Missing value patterns in % (sample size = 14,090)**		
SES		12.3
Grade German		4.1
Grade math		4.6
Grade physics		13.2
Grade chemistry		13.7
Grade biology		18.7
BMI		15.5
Test score German		1.4
Test score math		4.1
Test score science		4.4
Migration status		0.8
**Students reporting not getting a grade in a subject**		
		Analytical sample size
German	85	14,005
Math	126	13,964
Physics	1,134	12,956
Chemistry	1,192	12,898
Biology	1,883	12,207

Students from special needs schools are excluded.

Source: NEPS SC4, our own calculations.
