# Supplementary material for: Does chubby Can get lower grades than skinny Sophie? Using an intersectional approach to uncover grading bias in German secondary schools
Source: PLoS One. 2024 Jul 3;19(7):e0305703. doi: 10.1371/journal.pone.0305703 (PMC11221685; doi:10.1371/journal.pone.0305703)
Supplement: S2 Table — (PDF) [file pone.0305703.s011.pdf]

Table S2: Multilevel-linear regression results (regression coefficients and [95% confidence intervals]) predicting school Grades in German (models 1 + 2).

	Model 1	Model 1	Model 1	Model 1	Model 1	Model 2	Model 2
Gender (ref: boy)							
Girl	0.37***					0.29***	0.29***
	[0.33,0.41]					[0.24,0.33]	[0.24,0.33]
BMI (ref: non-overweight/obese)							
Overweight/obese		-0.21***				-0.10*	-0.10*
		[-0.28,-0.13]				[-0.17,-0.02]	[-0.17,-0.02]
SES (z)			0.08***			0.08***	0.07***
			[0.06,0.10]			[0.05,0.10]	[0.05,0.09]
Minority status / group (ref: majority)							
Minority				-0.14***		-0.12***	
				[-0.19,-0.09]		[-0.16,-0.07]	
Turkey					-0.24***		-0.22***
					[-0.33,-0.14]		[-0.31,-0.13]
FSU					-0.19***		-0.16**
					[-0.30,-0.09]		[-0.26,-0.06]
NW+South Europe					-0.10		-0.09
					[-0.21,0.01]		[-0.19,0.01]

Continued on the next page

Table S2: Continuation from the previous page

	Model 1	Model 1	Model 1	Model 1	Model 1	Model 2	Model 2
Central-Eastern Europe					-0.06		-0.05
					[-0.14,0.02]		[-0.13,0.03]
Other					-0.15***		-0.12**
					[-0.23,-0.06]		[-0.19,-0.04]
Test score	0.27***	0.30***	0.29***	0.29***	0.29***	0.25***	0.25***
	[0.25,0.30]	[0.27,0.32]	[0.26,0.31]	[0.26,0.32]	[0.26,0.31]	[0.23,0.28]	[0.23,0.28]
Reasoning score	0.00	-0.02	-0.02	-0.03*	-0.03*	0.00	0.00
	[-0.03,0.03]	[-0.05,0.00]	[-0.05,0.00]	[-0.05,-0.00]	[-0.05,-0.00]	[-0.02,0.03]	[-0.02,0.03]
Perceptual speed score	0.03*	0.06***	0.07***	0.07***	0.07***	0.03*	0.03*
	[0.00,0.05]	[0.04,0.09]	[0.04,0.09]	[0.04,0.09]	[0.04,0.09]	[0.00,0.05]	[0.00,0.05]
School type (ref: *Hauptschule*)							
SmmB	0.04	0.05	0.04	0.04	0.04	0.01	0.01
	[-0.06,0.15]	[-0.05,0.16]	[-0.07,0.14]	[-0.06,0.15]	[-0.07,0.14]	[-0.09,0.11]	[-0.09,0.11]
Realschule	-0.16***	-0.15***	-0.16***	-0.15***	-0.15***	-0.20***	-0.21***
	[-0.25,-0.07]	[-0.24,-0.06]	[-0.25,-0.08]	[-0.24,-0.06]	[-0.24,-0.06]	[-0.29,-0.12]	[-0.29,-0.12]
Gymnasium	-0.02	-0.01	-0.06	-0.00	-0.01	-0.12*	-0.12*
	[-0.11,0.07]	[-0.10,0.08]	[-0.15,0.04]	[-0.09,0.09]	[-0.10,0.08]	[-0.21,-0.03]	[-0.21,-0.03]

Continued on the next page

Table S2: Continuation from the previous page

	Model 1	Model 1	Model 1	Model 1	Model 1	Model 2	Model 2
SDQ: Prosocial (z)						0.04***	0.04***
						[0.02,0.07]	[0.02,0.07]
SDQ: Problems (z)						0.01	0.01
						[-0.02,0.03]	[-0.02,0.03]
SCOFF score						-0.02*	-0.02*
						[-0.04,-0.00]	[-0.04,-0.00]
Health satisf. (z)						-0.02	-0.02
						[-0.04,0.00]	[-0.04,0.01]
Class retention (ref: no)						-0.18***	-0.18***
						[-0.24,-0.13]	[-0.24,-0.13]
Neuroticism (z)						0.00	0.00
						[-0.02,0.03]	[-0.02,0.02]
Openness (z)						0.01	0.01
						[-0.01,0.03]	[-0.01,0.03]
Extraversion (z)						0.06***	0.06***
						[0.04,0.08]	[0.04,0.08]
Agreeableness (z)						-0.04***	-0.04***
						[-0.06,-0.02]	[-0.06,-0.02]

Continued on the next page

Table S2: Continuation from the previous page

	Model 1	Model 1	Model 1	Model 1	Model 1	Model 2	Model 2
Conscientiousness (z)						0.15***	0.15***
						[0.12,0.17]	[0.12,0.17]
Intercept	-0.17***	0.03	0.03	0.04	0.05	0.04	0.04
	[-0.23,-0.10]	[-0.03,0.10]	[-0.03,0.10]	[-0.02,0.11]	[-0.02,0.11]	[-0.04,0.11]	[-0.03,0.11]
SD(school)	0.19***	0.19***	0.19***	0.19***	0.19***	0.18***	0.19***
	[0.15,0.25]	[0.15,0.25]	[0.15,0.25]	[0.15,0.25]	[0.15,0.25]	[0.14,0.24]	[0.14,0.24]
SD(class)	0.29***	0.30***	0.30***	0.30***	0.30***	0.28***	0.28***
	[0.26,0.34]	[0.26,0.34]	[0.26,0.34]	[0.26,0.34]	[0.26,0.34]	[0.25,0.32]	[0.25,0.32]
Sigma	0.87***	0.89***	0.89***	0.89***	0.89***	0.85***	0.85***
	[0.86,0.89]	[0.87,0.90]	[0.87,0.90]	[0.87,0.90]	[0.87,0.90]	[0.83,0.86]	[0.83,0.86]
N	14005	14005	14005	14005	14005	14005	14005

Note: ***p≤0.001, **p≤0.01, *p≤0.05

Source: NEPS SC4 (based on m = 50 multiple imputed datasets); weighted data, our own calculations.
