# Supplementary material for: Does chubby Can get lower grades than skinny Sophie? Using an intersectional approach to uncover grading bias in German secondary schools
Source: PLoS One. 2024 Jul 3;19(7):e0305703. doi: 10.1371/journal.pone.0305703 (PMC11221685; doi:10.1371/journal.pone.0305703)
Supplement: S3 Table — (PDF) [file pone.0305703.s012.pdf]

Table S3: Multilevel-linear regression results (regression coefficients and [95% confidence intervals]) predicting school Grades in Math (models 1 + 2).

	Model 1	Model 1	Model 1	Model 1	Model 1	Model 2	Model 2
Gender (ref: boy)							
Girl	0.06**					-0.01	-0.01
	[0.02,0.11]					[-0.06,0.04]	[-0.06,0.04]
BMI (ref: non-overweight/obese)							
Overweight/obese		-0.07*				0.00	0.00
		[-0.13,-0.01]				[-0.06,0.06]	[-0.06,0.07]
SES (z)			0.04***			0.05***	0.05***
			[0.02,0.06]			[0.03,0.07]	[0.03,0.07]
Minority status / group (ref: majority)							
Minority				-0.08**		-0.04	
				[-0.12,-0.03]		[-0.09,0.00]	
Turkey					-0.08		-0.08
					[-0.16,0.01]		[-0.16,0.01]
FSU					0.01		0.06
					[-0.08,0.09]		[-0.03,0.14]
NW+South Europe					-0.06		-0.03
					[-0.16,0.04]		[-0.13,0.06]

Continued on the next page

Table S3: Continuation from the previous page

	Model 1	Model 1	Model 1	Model 1	Model 1	Model 2	Model 2
Central-Eastern Europe					-0.11**		-0.08*
					[-0.19,-0.03]		[-0.15,-0.00]
Other					-0.12**		-0.06
					[-0.19,-0.04]		[-0.13,0.00]
Test score	0.45***	0.44***	0.43***	0.43***	0.43***	0.42***	0.42***
	[0.42,0.47]	[0.41,0.46]	[0.40,0.46]	[0.40,0.46]	[0.40,0.46]	[0.39,0.45]	[0.39,0.45]
Reasoning score	0.12***	0.12***	0.12***	0.12***	0.12***	0.13***	0.13***
	[0.09,0.15]	[0.09,0.15]	[0.09,0.15]	[0.09,0.15]	[0.09,0.14]	[0.10,0.15]	[0.10,0.15]
Perceptual speed score	0.07***	0.07***	0.07***	0.07***	0.07***	0.07***	0.07***
	[0.04,0.09]	[0.05,0.10]	[0.05,0.10]	[0.05,0.10]	[0.05,0.10]	[0.04,0.09]	[0.04,0.09]
School type (ref: *Hauptschule*)							
SmmB	-0.11**	-0.11**	-0.12**	-0.12**	-0.11**	-0.11**	-0.11**
	[-0.19,-0.03]	[-0.19,-0.03]	[-0.20,-0.04]	[-0.20,-0.04]	[-0.19,-0.03]	[-0.19,-0.03]	[-0.18,-0.03]
Realschule	-0.29***	-0.29***	-0.30***	-0.29***	-0.29***	-0.30***	-0.30***
	[-0.38,-0.21]	[-0.37,-0.21]	[-0.38,-0.21]	[-0.38,-0.21]	[-0.37,-0.21]	[-0.39,-0.22]	[-0.39,-0.22]
Gymnasium	-0.55***	-0.54***	-0.57***	-0.54***	-0.54***	-0.57***	-0.56***
	[-0.64,-0.47]	[-0.62,-0.46]	[-0.65,-0.49]	[-0.62,-0.46]	[-0.62,-0.45]	[-0.65,-0.48]	[-0.65,-0.48]

Continued on the next page

Table S3: Continuation from the previous page

	Model 1	Model 1	Model 1	Model 1	Model 1	Model 2	Model 2
SDQ: Prosocial (z)						0.04***	0.04***
						[0.02,0.07]	[0.02,0.07]
SDQ: Problems (z)						-0.00	-0.00
						[-0.02,0.02]	[-0.02,0.02]
SCOFF score						-0.02	-0.02
						[-0.04,0.00]	[-0.04,0.00]
Health satisf. (z)						0.03**	0.03**
						[0.01,0.05]	[0.01,0.05]
Class retention (ref: no)						-0.17***	-0.17***
						[-0.22,-0.11]	[-0.23,-0.11]
Neuroticism (z)						-0.01	-0.01
						[-0.03,0.01]	[-0.03,0.01]
Openness (z)						-0.05***	-0.05***
						[-0.07,-0.03]	[-0.07,-0.03]
Extraversion (z)						-0.04***	-0.04***
						[-0.06,-0.02]	[-0.06,-0.02]
Agreeableness (z)						-0.00	-0.00
						[-0.02,0.02]	[-0.02,0.02]

Continued on the next page

Table S3: Continuation from the previous page

	Model 1	Model 1	Model 1	Model 1	Model 1	Model 2	Model 2
Conscientiousness (z)						0.18***	0.18***
						[0.16,0.20]	[0.16,0.20]
Intercept	0.24***	0.27***	0.28***	0.29***	0.28***	0.35***	0.35***
	[0.18,0.30]	[0.21,0.33]	[0.22,0.34]	[0.23,0.34]	[0.23,0.34]	[0.28,0.42]	[0.28,0.42]
SD(school)	0.17***	0.17***	0.17***	0.17***	0.17***	0.16***	0.16***
	[0.13,0.22]	[0.13,0.22]	[0.13,0.22]	[0.13,0.22]	[0.13,0.22]	[0.12,0.21]	[0.12,0.21]
SD(class)	0.23***	0.23***	0.23***	0.23***	0.23***	0.23***	0.23***
	[0.20,0.26]	[0.20,0.27]	[0.20,0.27]	[0.20,0.27]	[0.20,0.26]	[0.20,0.27]	[0.20,0.26]
Sigma	0.87***	0.87***	0.87***	0.87***	0.87***	0.85***	0.85***
	[0.86,0.89]	[0.86,0.89]	[0.86,0.89]	[0.86,0.89]	[0.86,0.89]	[0.83,0.86]	[0.83,0.86]
N	13964	13964	13964	13964	13964	13964	13964

Note: ***p≤0.001, **p≤0.01, *p≤0.05

Source: NEPS SC4 (based on m = 50 multiple imputed datasets); weighted data, our own calculations.
