# Supplementary material for: Does chubby Can get lower grades than skinny Sophie? Using an intersectional approach to uncover grading bias in German secondary schools
Source: PLoS One. 2024 Jul 3;19(7):e0305703. doi: 10.1371/journal.pone.0305703 (PMC11221685; doi:10.1371/journal.pone.0305703)
Supplement: S4 Table — (PDF) [file pone.0305703.s013.pdf]

Table S4: Multilevel-linear regression results (regression coefficients and [95% confidence intervals]) predicting school Grades in Physics (models 1 + 2).

	Model 1	Model 1	Model 1	Model 1	Model 1	Model 2	Model 2
Gender (ref: boy)							
Girl	-0.10***					-0.20***	-0.20***
	[-0.14,-0.05]					[-0.24,-0.15]	[-0.24,-0.15]
BMI (ref: non-overweight/obese)							
Overweight/obese		-0.07*				-0.05	-0.05
		[-0.14,-0.01]				[-0.12,0.01]	[-0.12,0.01]
SES (z)			0.06***			0.06***	0.06***
			[0.03,0.08]			[0.03,0.08]	[0.03,0.09]
Minority status / group (ref: majority)							
Minority				-0.09***		-0.06*	
				[-0.15,-0.04]		[-0.11,-0.00]	
Turkey					-0.06		-0.06
					[-0.16,0.04]		[-0.15,0.04]
FSU					-0.05		0.00
					[-0.15,0.04]		[-0.10,0.10]
NW+South Europe					-0.13*		-0.11*
					[-0.24,-0.02]		[-0.22,-0.01]

Continued on the next page

Table S4: Continuation from the previous page

	Model 1	Model 1	Model 1	Model 1	Model 1	Model 2	Model 2
Central-Eastern Europe					-0.07		-0.03
					[-0.16,0.02]		[-0.12,0.06]
Other					-0.14***		-0.09*
					[-0.22,-0.06]		[-0.16,-0.01]
Test score	0.24***	0.25***	0.24***	0.24***	0.24***	0.23***	0.23***
	[0.21,0.27]	[0.22,0.28]	[0.21,0.27]	[0.21,0.27]	[0.21,0.27]	[0.21,0.26]	[0.21,0.26]
Reasoning score	0.08***	0.08***	0.08***	0.08***	0.08***	0.08***	0.08***
	[0.06,0.11]	[0.06,0.11]	[0.06,0.11]	[0.06,0.11]	[0.06,0.11]	[0.06,0.11]	[0.06,0.11]
Perceptual speed score	0.06***	0.05***	0.06***	0.06***	0.06***	0.06***	0.06***
	[0.04,0.09]	[0.03,0.08]	[0.03,0.08]	[0.03,0.08]	[0.03,0.08]	[0.04,0.09]	[0.04,0.09]
School type (ref: *Hauptschule*)							
SmmB	-0.15**	-0.16**	-0.17**	-0.17**	-0.16**	-0.15**	-0.15**
	[-0.25,-0.04]	[-0.26,-0.05]	[-0.28,-0.07]	[-0.27,-0.06]	[-0.27,-0.06]	[-0.25,-0.05]	[-0.25,-0.05]
Realschule	-0.25***	-0.26***	-0.28***	-0.26***	-0.26***	-0.27***	-0.28***
	[-0.34,-0.15]	[-0.36,-0.17]	[-0.37,-0.19]	[-0.36,-0.17]	[-0.36,-0.17]	[-0.37,-0.18]	[-0.37,-0.18]
Gymnasium	-0.20***	-0.23***	-0.27***	-0.23***	-0.23***	-0.25***	-0.25***
	[-0.30,-0.10]	[-0.33,-0.14]	[-0.37,-0.18]	[-0.32,-0.13]	[-0.32,-0.13]	[-0.35,-0.15]	[-0.35,-0.15]

Continued on the next page

Table S4: Continuation from the previous page

	Model 1	Model 1	Model 1	Model 1	Model 1	Model 2	Model 2
SDQ: Prosocial (z)						0.06***	0.06***
						[0.04,0.09]	[0.04,0.09]
SDQ: Problems (z)						0.02	0.02
						[-0.01,0.04]	[-0.01,0.04]
SCOFF score						-0.01	-0.01
						[-0.03,0.01]	[-0.03,0.01]
Health satisf. (z)						0.01	0.01
						[-0.01,0.03]	[-0.01,0.03]
Class retention (ref: no)						-0.20***	-0.20***
						[-0.26,-0.14]	[-0.26,-0.14]
Neuroticism (z)						-0.00	-0.00
						[-0.02,0.02]	[-0.02,0.02]
Openness (z)						-0.02	-0.02
						[-0.04,0.01]	[-0.04,0.01]
Extraversion (z)						0.00	0.00
						[-0.02,0.02]	[-0.02,0.02]
Agreeableness (z)						-0.02	-0.02
						[-0.04,0.00]	[-0.04,0.00]

Continued on the next page

Table S4: Continuation from the previous page

	Model 1	Model 1	Model 1	Model 1	Model 1	Model 2	Model 2
Conscientiousness (z)						0.19***	0.19***
						[0.16,0.21]	[0.16,0.21]
Intercept	0.18***	0.17***	0.18***	0.18***	0.18***	0.34***	0.34***
	[0.12,0.25]	[0.10,0.23]	[0.11,0.24]	[0.11,0.25]	[0.11,0.25]	[0.27,0.41]	[0.27,0.41]
SD(school)	0.27***	0.27***	0.26***	0.27***	0.27***	0.26***	0.26***
	[0.24,0.30]	[0.23,0.30]	[0.23,0.30]	[0.24,0.30]	[0.24,0.30]	[0.23,0.29]	[0.23,0.30]
SD(class)	0.20***	0.20***	0.20***	0.20***	0.20***	0.20***	0.20***
	[0.17,0.24]	[0.17,0.24]	[0.17,0.24]	[0.17,0.24]	[0.17,0.24]	[0.17,0.24]	[0.17,0.24]
Sigma	0.90***	0.90***	0.90***	0.90***	0.90***	0.87***	0.87***
	[0.88,0.91]	[0.88,0.91]	[0.88,0.91]	[0.88,0.91]	[0.88,0.91]	[0.85,0.88]	[0.85,0.88]
N	12956	12956	12956	12956	12956	12956	12956

Note: ***p≤0.001, **p≤0.01, *p≤0.05

Source: NEPS SC4 (based on m = 50 multiple imputed datasets); weighted data, our own calculations.
