# Supplementary material for: Does chubby Can get lower grades than skinny Sophie? Using an intersectional approach to uncover grading bias in German secondary schools
Source: PLoS One. 2024 Jul 3;19(7):e0305703. doi: 10.1371/journal.pone.0305703 (PMC11221685; doi:10.1371/journal.pone.0305703)
Supplement: S5 Table — (PDF) [file pone.0305703.s014.pdf]

Table S5: Multilevel-linear regression results (regression coefficients and [95% confidence intervals]) predicting school Grades in Chemistry (models 1 + 2).

	Model 1	Model 1	Model 1	Model 1	Model 1	Model 2	Model 2
Gender (ref: boy)							
Girl	0.02					-0.09***	-0.09***
	[-0.03,0.07]					[-0.14,-0.04]	[-0.14,-0.04]
BMI (ref: non-overweight/obese)							
Overweight/obese		-0.08*				-0.04	-0.04
		[-0.15,-0.01]				[-0.11,0.03]	[-0.11,0.03]
SES (z)			0.04***			0.04***	0.04***
			[0.02,0.07]			[0.02,0.07]	[0.02,0.07]
Minority status / group (ref: majority)							
Minority				-0.07**		-0.04	
				[-0.13,-0.02]		[-0.09,0.01]	
Turkey					-0.04		-0.03
					[-0.14,0.05]		[-0.12,0.06]
FSU					-0.04		0.01
					[-0.14,0.06]		[-0.09,0.10]
NW+South Europe					-0.11*		-0.09
					[-0.22,-0.01]		[-0.19,0.01]

Continued on the next page

Table S5: Continuation from the previous page

	Model 1	Model 1	Model 1	Model 1	Model 1	Model 2	Model 2
Central-Eastern Europe					-0.10*		-0.07
					[-0.19,-0.01]		[-0.16,0.02]
Other					-0.06		-0.01
					[-0.14,0.02]		[-0.09,0.06]
Test score	0.25***	0.25***	0.25***	0.25***	0.25***	0.25***	0.25***
	[0.23,0.28]	[0.23,0.28]	[0.22,0.27]	[0.22,0.27]	[0.22,0.27]	[0.23,0.28]	[0.23,0.28]
Reasoning score	0.07***	0.07***	0.07***	0.07***	0.07***	0.07***	0.07***
	[0.04,0.10]	[0.04,0.10]	[0.04,0.10]	[0.04,0.10]	[0.04,0.10]	[0.05,0.10]	[0.05,0.10]
Perceptual speed score	0.06***	0.07***	0.07***	0.07***	0.07***	0.06***	0.06***
	[0.04,0.09]	[0.04,0.09]	[0.04,0.09]	[0.04,0.09]	[0.04,0.09]	[0.04,0.09]	[0.04,0.09]
School type (ref: *Hauptschule*)							
SmmB	-0.13**	-0.13**	-0.14**	-0.13**	-0.13**	-0.13**	-0.13**
	[-0.23,-0.03]	[-0.23,-0.03]	[-0.23,-0.04]	[-0.23,-0.04]	[-0.23,-0.04]	[-0.22,-0.03]	[-0.22,-0.04]
Realschule	-0.21***	-0.21***	-0.22***	-0.21***	-0.21***	-0.23***	-0.23***
	[-0.31,-0.11]	[-0.31,-0.11]	[-0.32,-0.12]	[-0.31,-0.11]	[-0.31,-0.11]	[-0.33,-0.13]	[-0.33,-0.13]
Gymnasium	-0.22***	-0.23***	-0.26***	-0.22***	-0.22***	-0.27***	-0.27***
	[-0.32,-0.12]	[-0.33,-0.13]	[-0.36,-0.15]	[-0.32,-0.12]	[-0.32,-0.12]	[-0.37,-0.16]	[-0.37,-0.16]

Continued on the next page

Table S5: Continuation from the previous page

	Model 1	Model 1	Model 1	Model 1	Model 1	Model 2	Model 2
SDQ: Prosocial (z)						0.07***	0.07***
						[0.05,0.10]	[0.05,0.10]
SDQ: Problems (z)						0.02	0.02
						[-0.01,0.04]	[-0.01,0.04]
SCOFF score						-0.01	-0.01
						[-0.03,0.01]	[-0.03,0.01]
Health satisf. (z)						0.01	0.01
						[-0.01,0.04]	[-0.01,0.04]
Class retention (ref: no)						-0.23***	-0.23***
						[-0.29,-0.18]	[-0.29,-0.18]
Neuroticism (z)						-0.02	-0.02
						[-0.04,0.00]	[-0.04,0.00]
Openness (z)						-0.01	-0.01
						[-0.03,0.01]	[-0.03,0.01]
Extraversion (z)						-0.00	-0.00
						[-0.02,0.02]	[-0.02,0.02]
Agreeableness (z)						-0.02	-0.02
						[-0.04,0.00]	[-0.04,0.00]

Continued on the next page

Table S5: Continuation from the previous page

	Model 1	Model 1	Model 1	Model 1	Model 1	Model 2	Model 2
Conscientiousness (z)						0.18***	0.18***
						[0.15,0.20]	[0.15,0.20]
Intercept	0.12***	0.14***	0.14***	0.15***	0.15***	0.27***	0.27***
	[0.05,0.18]	[0.07,0.21]	[0.08,0.21]	[0.08,0.21]	[0.08,0.21]	[0.19,0.35]	[0.19,0.35]
SD(school)	0.26***	0.26***	0.26***	0.26***	0.26***	0.26***	0.26***
	[0.22,0.30]	[0.22,0.30]	[0.22,0.30]	[0.22,0.30]	[0.22,0.30]	[0.22,0.30]	[0.22,0.30]
SD(class)	0.22***	0.22***	0.22***	0.23***	0.23***	0.20***	0.21***
	[0.19,0.26]	[0.19,0.26]	[0.19,0.26]	[0.19,0.26]	[0.19,0.26]	[0.17,0.24]	[0.17,0.24]
Sigma	0.90***	0.90***	0.90***	0.90***	0.90***	0.87***	0.87***
	[0.88,0.91]	[0.88,0.91]	[0.88,0.91]	[0.88,0.91]	[0.88,0.91]	[0.85,0.88]	[0.85,0.88]
N	12898	12898	12898	12898	12898	12898	12898

Note: ***p≤0.001, **p≤0.01, *p≤0.05

Source: NEPS SC4 (based on m = 50 multiple imputed datasets); weighted data, our own calculations.
