# Supplementary material for: Does chubby Can get lower grades than skinny Sophie? Using an intersectional approach to uncover grading bias in German secondary schools
Source: PLoS One. 2024 Jul 3;19(7):e0305703. doi: 10.1371/journal.pone.0305703 (PMC11221685; doi:10.1371/journal.pone.0305703)
Supplement: S6 Table — (PDF) [file pone.0305703.s015.pdf]

Table S6: Multilevel-linear regression results (regression coefficients and [95% confidence intervals]) predicting school Grades in Biology (models 1 + 2).

	Model 1	Model 1	Model 1	Model 1	Model 1	Model 2	Model 2
Gender (ref: boy)							
Girl	0.21***					0.08***	0.08***
	[0.16,0.26]					[0.04,0.13]	[0.04,0.13]
BMI (ref: non-overweight/obese)							
Overweight/obese		-0.12**				-0.04	-0.04
		[-0.19,-0.05]				[-0.11,0.04]	[-0.11,0.04]
SES (z)			0.06***			0.06***	0.06***
			[0.04,0.09]			[0.04,0.09]	[0.04,0.09]
Minority status / group (ref: majority)							
Minority				-0.04		-0.01	
				[-0.09,0.01]		[-0.06,0.04]	
Turkey					-0.05		-0.04
					[-0.15,0.05]		[-0.14,0.06]
FSU					-0.05		0.01
					[-0.15,0.04]		[-0.09,0.10]
NW+South Europe					-0.06		-0.04
					[-0.19,0.07]		[-0.16,0.09]

Continued on the next page

Table S6: Continuation from the previous page

	Model 1	Model 1	Model 1	Model 1	Model 1	Model 2	Model 2
Central-Eastern Europe					-0.01		0.02
					[-0.09,0.08]		[-0.06,0.10]
Other					-0.05		-0.01
					[-0.14,0.04]		[-0.09,0.08]
Test score	0.24***	0.22***	0.21***	0.22***	0.22***	0.24***	0.24***
	[0.21,0.27]	[0.20,0.25]	[0.19,0.24]	[0.19,0.25]	[0.19,0.25]	[0.21,0.27]	[0.21,0.27]
Reasoning score	0.05***	0.04**	0.04**	0.04**	0.04**	0.05***	0.05***
	[0.02,0.07]	[0.01,0.06]	[0.02,0.07]	[0.01,0.06]	[0.01,0.06]	[0.03,0.07]	[0.03,0.07]
Perceptual speed score	0.05***	0.07***	0.07***	0.07***	0.07***	0.05***	0.05***
	[0.02,0.07]	[0.04,0.09]	[0.05,0.10]	[0.04,0.09]	[0.04,0.10]	[0.02,0.07]	[0.02,0.07]
School type (ref: *Hauptschule*)							
SmmB	-0.04	-0.02	-0.03	-0.02	-0.02	-0.04	-0.04
	[-0.14,0.07]	[-0.13,0.08]	[-0.14,0.07]	[-0.13,0.08]	[-0.13,0.08]	[-0.14,0.06]	[-0.14,0.06]
Realschule	-0.07	-0.06	-0.07	-0.05	-0.05	-0.10	-0.10
	[-0.18,0.03]	[-0.16,0.04]	[-0.18,0.03]	[-0.16,0.05]	[-0.16,0.05]	[-0.20,0.00]	[-0.20,0.00]
Gymnasium	-0.04	-0.01	-0.05	0.00	0.00	-0.11*	-0.11*
	[-0.14,0.05]	[-0.10,0.09]	[-0.15,0.05]	[-0.09,0.10]	[-0.09,0.10]	[-0.20,-0.01]	[-0.20,-0.01]

Continued on the next page

Table S6: Continuation from the previous page

	Model 1	Model 1	Model 1	Model 1	Model 1	Model 2	Model 2
SDQ: Prosocial (z)						0.07***	0.07***
						[0.04,0.10]	[0.05,0.10]
SDQ: Problems (z)						0.01	0.01
						[-0.01,0.03]	[-0.01,0.03]
SCOFF score						0.00	0.01
						[-0.02,0.03]	[-0.02,0.03]
Health satisf. (z)						0.01	0.01
						[-0.01,0.04]	[-0.01,0.04]
Class retention (ref: no)						-0.21***	-0.21***
						[-0.27,-0.15]	[-0.27,-0.15]
Neuroticism (z)						-0.00	-0.00
						[-0.03,0.02]	[-0.03,0.02]
Openness (z)						0.00	0.00
						[-0.02,0.02]	[-0.02,0.02]
Extraversion (z)						0.04***	0.04***
						[0.02,0.06]	[0.02,0.06]
Agreeableness (z)						-0.03*	-0.03*
						[-0.05,-0.00]	[-0.05,-0.00]

Continued on the next page

Table S6: Continuation from the previous page

	Model 1	Model 1	Model 1	Model 1	Model 1	Model 2	Model 2
Conscientiousness (z)						0.20***	0.20***
						[0.18,0.23]	[0.18,0.23]
Intercept	-0.08*	0.02	0.02	0.01	0.01	0.06	0.06
	[-0.15,-0.01]	[-0.05,0.08]	[-0.04,0.09]	[-0.06,0.07]	[-0.06,0.07]	[-0.01,0.14]	[-0.01,0.14]
SD(school)	0.22***	0.22***	0.22***	0.22***	0.22***	0.21***	0.21***
	[0.17,0.27]	[0.18,0.28]	[0.18,0.27]	[0.18,0.27]	[0.18,0.27]	[0.16,0.26]	[0.16,0.26]
SD(class)	0.28***	0.28***	0.28***	0.28***	0.28***	0.27***	0.27***
	[0.23,0.33]	[0.23,0.33]	[0.23,0.33]	[0.24,0.33]	[0.24,0.33]	[0.23,0.32]	[0.23,0.32]
Sigma	0.89***	0.90***	0.90***	0.90***	0.90***	0.86***	0.86***
	[0.87,0.91]	[0.87,0.92]	[0.87,0.92]	[0.88,0.92]	[0.88,0.92]	[0.84,0.88]	[0.84,0.88]
N	12207	12207	12207	12207	12207	12207	12207

Note: ***p≤0.001, **p≤0.01, *p≤0.05

Source: NEPS SC4 (based on m = 50 multiple imputed datasets); weighted data, our own calculations.
