# Supplementary material for: Does chubby Can get lower grades than skinny Sophie? Using an intersectional approach to uncover grading bias in German secondary schools
Source: PLoS One. 2024 Jul 3;19(7):e0305703. doi: 10.1371/journal.pone.0305703 (PMC11221685; doi:10.1371/journal.pone.0305703)
Supplement: S7 Table — (PDF) [file pone.0305703.s016.pdf]

Table S7: Multilevel-linear regression results (regression coefficients and [95% confidence intervals]) predicting school Grades in German (Intersectional models).

	Model no IE	Model 2-way IE	Model 4-way IE	Model no IE	Model 2-way IE	Model 4-way IE
Gender (ref: boy)						
Girl	0.37***	0.37***	0.37***	0.37***	0.37***	0.37***
	[0.32,0.41]	[0.31,0.42]	[0.33,0.41]	[0.32,0.41]	[0.31,0.42]	[0.33,0.42]
BMI (ref: non-overweight/obese)						
Overweight/obese	-0.12**	-0.12*	-0.13***	-0.12**	-0.12*	-0.13***
	[-0.20,-0.05]	[-0.22,-0.01]	[-0.21,-0.05]	[-0.20,-0.05]	[-0.22,-0.01]	[-0.21,-0.05]
SES (z)	0.08***	0.04*	0.04	0.08***	0.04*	0.04
	[0.05,0.10]	[0.00,0.07]	[-0.00,0.07]	[0.05,0.10]	[0.00,0.07]	[-0.00,0.08]
Minority status / group (ref: majority)						
Minority	-0.13***	-0.15***	-0.13***			
	[-0.18,-0.09]	[-0.23,-0.07]	[-0.18,-0.09]			
Turkey				-0.22***	-0.19*	-0.25***
				[-0.31,-0.12]	[-0.35,-0.04]	[-0.38,-0.13]
FSU				-0.18***	-0.20*	-0.17**
				[-0.28,-0.08]	[-0.35,-0.04]	[-0.27,-0.06]
NW+South Europe				-0.10*	-0.13	-0.10
				[-0.21,-0.00]	[-0.31,0.05]	[-0.21,0.00]

Continued on the next page

Table S7: Continuation from the previous page

	Model no IE	Model 2-way IE	Model 4-way IE	Model no IE	Model 2-way IE	Model 4-way IE
Central-Eastern Europe				-0.07	-0.17*	-0.06
				[-0.15,0.02]	[-0.30,-0.03]	[-0.15,0.02]
Other				-0.14***	-0.11	-0.14***
				[-0.22,-0.06]	[-0.25,0.03]	[-0.22,-0.06]
Test score	0.26***	0.26***	0.26***	0.26***	0.26***	0.26***
	[0.23,0.28]	[0.23,0.28]	[0.23,0.28]	[0.23,0.28]	[0.23,0.28]	[0.23,0.28]
Reasoning score	-0.00	-0.00	-0.00	-0.00	-0.00	-0.00
	[-0.03,0.02]	[-0.03,0.02]	[-0.03,0.02]	[-0.03,0.02]	[-0.03,0.02]	[-0.03,0.02]
Perceptual speed score	0.03**	0.03**	0.03**	0.03**	0.03**	0.03**
	[0.01,0.06]	[0.01,0.06]	[0.01,0.06]	[0.01,0.06]	[0.01,0.06]	[0.01,0.06]
School type (ref: *Hauptschule*)						
SmmB	0.00	0.00	0.00	0.00	0.00	0.00
	[-0.10,0.11]	[-0.10,0.11]	[-0.10,0.11]	[-0.10,0.10]	[-0.10,0.10]	[-0.10,0.10]
Realschule	-0.20***	-0.20***	-0.20***	-0.21***	-0.20***	-0.21***
	[-0.29,-0.12]	[-0.29,-0.11]	[-0.29,-0.11]	[-0.29,-0.12]	[-0.29,-0.12]	[-0.29,-0.12]
Gymnasium	-0.11*	-0.11*	-0.11*	-0.11*	-0.12*	-0.12*
	[-0.20,-0.02]	[-0.20,-0.02]	[-0.20,-0.02]	[-0.21,-0.02]	[-0.21,-0.02]	[-0.21,-0.03]

Continued on the next page

Table S7: Continuation from the previous page

	Model no IE	Model 2-way IE	Model 4-way IE	Model no IE	Model 2-way IE	Model 4-way IE
Interactions						
Minority x overweight/obese		-0.02				
		[-0.16,0.12]				
Minority x girl		0.03				
		[-0.07,0.13]				
Minority x SES (z)		0.02				
		[-0.03,0.06]				
Overweight/obese x girl		-0.03			-0.03	
		[-0.18,0.11]			[-0.18,0.12]	
Overweight/obese x SES (z)		-0.03			-0.03	
		[-0.10,0.04]			[-0.10,0.04]	
Girl x SES (z)		0.08***			0.08***	
		[0.04,0.12]			[0.04,0.12]	
Majority x non-overweight/obese x girl x SES (z)			0.08**			
			[0.03,0.13]			
Majority x overweight/obese x boy x SES (z)			-0.04			
			[-0.14,0.07]			

Continued on the next page

Table S7: Continuation from the previous page

	Model no IE	Model 2-way IE	Model 4-way IE	Model no IE	Model 2-way IE	Model 4-way IE
Majority x overweight/obese x girl x SES (z)			0.09			
			[-0.07,0.25]			
Minority x non-overweight/obese x boy x SES (z)			0.03			
			[-0.05,0.10]			
Minority x non-overweight/obese x girl x SES (z)			0.09**			
			[0.03,0.16]			
Minority x overweight/obese x boy x SES (z)			-0.01			
			[-0.15,0.13]			
Minority x overweight/obese x girl x SES (z)			0.05			
			[-0.12,0.22]			
Turkey x overweight/obese					-0.01	
					[-0.26,0.25]	
FSU x overweight/obese					0.13	
					[-0.15,0.41]	
NW+South Europe x overweight/obese					-0.00	
					[-0.34,0.34]	
Central-Eastern Europe x overweight/obese					-0.09	
					[-0.33,0.16]	

Continued on the next page

Table S7: Continuation from the previous page

	Model no IE	Model 2-way IE	Model 4-way IE	Model no IE	Model 2-way IE	Model 4-way IE
Other x overweight/obese					-0.03	
					[-0.22,0.17]	
Turkey x girl					-0.10	
					[-0.28,0.08]	
FSU x girl					0.03	
					[-0.17,0.24]	
NW+South Europe x girl					0.05	
					[-0.17,0.26]	
Central-Eastern Europe x girl					0.22**	
					[0.06,0.38]	
Other x girl					-0.06	
					[-0.24,0.13]	
Turkey x SES (z)					-0.04	
					[-0.15,0.07]	
FSU x SES (z)					0.04	
					[-0.06,0.14]	
NW+South Europe x SES (z)					0.04	
					[-0.06,0.15]	

Continued on the next page

Table S7: Continuation from the previous page

	Model no IE	Model 2-way IE	Model 4-way IE	Model no IE	Model 2-way IE	Model 4-way IE
Central-Eastern Europe x SES (z)					0.01	
					[-0.08,0.09]	
Other x SES (z)					-0.00	
					[-0.08,0.07]	
Majority x non-overweight/obese x girl x SES (z)						0.08**
						[0.03,0.13]
Majority x overweight/obese x boy x SES (z)						-0.04
						[-0.14,0.07]
Majority x overweight/obese x girl x SES (z)						0.09
						[-0.07,0.25]
Turkey x non-overweight/obese x boy x SES (z)						-0.03
						[-0.18,0.11]
Turkey x non-overweight/obese x girl x SES (z)						0.05
						[-0.10,0.19]
Turkey x overweight/obese x boy x SES (z)						-0.08
						[-0.38,0.22]
Turkey x overweight/obese x girl x SES (z)						-0.08
						[-0.33,0.17]

Continued on the next page

Table S7: Continuation from the previous page

	Model no IE	Model 2-way IE	Model 4-way IE	Model no IE	Model 2-way IE	Model 4-way IE
FSU x non-overweight/obese x boy x SES (z)						-0.03
						[-0.18,0.13]
FSU x non-overweight/obese x girl x SES (z)						0.18**
						[0.05,0.30]
FSU x overweight/obese x boy x SES (z)						0.00
						[-0.25,0.26]
FSU x overweight/obese x girl x SES (z)						-0.04
						[-0.59,0.50]
NW+South Europe x non-overweight/obese x boy x SES (z)						0.02
						[-0.14,0.18]
NW+South Europe x non-overweight/obese x girl x SES (z)						0.13
						[-0.00,0.27]
NW+South Europe x overweight/obese x boy x SES (z)						0.14
						[-0.25,0.52]
NW+South Europe x overweight/obese x girl x SES (z)						0.06
						[-0.53,0.65]
Central-Eastern Europe x non-overweight/obese x boy x SES (z)						0.04
						[-0.11,0.19]

Continued on the next page

Table S7: Continuation from the previous page

	Model no IE	Model 2-way IE	Model 4-way IE	Model no IE	Model 2-way IE	Model 4-way IE
Central-Eastern Europe x non-overweight/obese x girl x SES (z)						0.07
						[-0.05,0.19]
Central-Eastern Europe x overweight/obese x boy x SES (z)						-0.09
						[-0.36,0.19]
Central-Eastern Europe x overweight/obese x girl x SES (z)						0.11
						[-0.33,0.56]
Other x non-overweight/obese x boy x SES (z)						0.06
						[-0.07,0.19]
Other x non-overweight/obese x girl x SES (z)						0.02
						[-0.09,0.14]
Other x overweight/obese x boy x SES (z)						-0.04
						[-0.34,0.27]
Other x overweight/obese x girl x SES (z)						0.08
						[-0.21,0.36]
Intercept	-0.06	-0.06	-0.06	-0.05	-0.05	-0.06
	[-0.13,0.01]	[-0.13,0.02]	[-0.13,0.01]	[-0.12,0.02]	[-0.13,0.02]	[-0.13,0.01]
SD(school)	0.19***	0.19***	0.19***	0.19***	0.19***	0.19***
	[0.14,0.25]	[0.14,0.25]	[0.14,0.25]	[0.14,0.25]	[0.14,0.25]	[0.14,0.25]

Continued on the next page

Table S7: Continuation from the previous page

	Model no IE	Model 2-way IE	Model 4-way IE	Model no IE	Model 2-way IE	Model 4-way IE
SD(class)	0.29***	0.29***	0.29***	0.29***	0.29***	0.29***
	[0.25,0.33]	[0.25,0.33]	[0.25,0.33]	[0.25,0.33]	[0.25,0.33]	[0.25,0.33]
Sigma	0.87***	0.87***	0.87***	0.87***	0.87***	0.87***
	[0.85,0.88]	[0.85,0.88]	[0.85,0.88]	[0.85,0.88]	[0.85,0.88]	[0.85,0.88]
N	14005	14005	14005	14005	14005	14005

Note: ***$p \leq 0.001$, **$p \leq 0.01$, *$p \leq 0.05$

Source: NEPS SC4 (based on m = 50 multiple imputed datasets); weighted data, our own calculations.
