# Supplementary material for: Does chubby Can get lower grades than skinny Sophie? Using an intersectional approach to uncover grading bias in German secondary schools
Source: PLoS One. 2024 Jul 3;19(7):e0305703. doi: 10.1371/journal.pone.0305703 (PMC11221685; doi:10.1371/journal.pone.0305703)
Supplement: S8 Table — (PDF) [file pone.0305703.s017.pdf]

Table S8: Multilevel-linear regression results (regression coefficients and [95% confidence intervals]) predicting school Grades in Math (Intersectional models).

	Model no IE	Model 2-way IE	Model 4-way IE	Model no IE	Model 2-way IE	Model 4-way IE
Gender (ref: boy)						
Girl	0.06*	0.09**	0.06*	0.06*	0.09**	0.06*
	[0.01,0.11]	[0.03,0.14]	[0.01,0.11]	[0.01,0.11]	[0.03,0.14]	[0.01,0.10]
BMI (ref: non-overweight/obese)						
Overweight/obese	-0.05	-0.06	-0.06	-0.05	-0.06	-0.06
	[-0.11,0.01]	[-0.15,0.02]	[-0.12,0.01]	[-0.11,0.01]	[-0.15,0.02]	[-0.12,0.01]
SES (z)	0.04***	0.01	-0.00	0.04***	0.01	-0.00
	[0.02,0.06]	[-0.03,0.04]	[-0.04,0.03]	[0.02,0.06]	[-0.03,0.04]	[-0.04,0.03]
Minority status / group (ref: majority)						
Minority	-0.07**	-0.04	-0.07**			
	[-0.11,-0.02]	[-0.11,0.04]	[-0.12,-0.02]			
Turkey				-0.05	-0.04	-0.08
				[-0.14,0.03]	[-0.20,0.11]	[-0.20,0.03]
FSU				0.02	0.13	0.02
				[-0.07,0.11]	[-0.02,0.28]	[-0.09,0.12]
NW+South Europe				-0.06	-0.08	-0.06
				[-0.15,0.04]	[-0.25,0.10]	[-0.16,0.04]

Continued on the next page

Table S8: Continuation from the previous page

	Model no IE	Model 2-way IE	Model 4-way IE	Model no IE	Model 2-way IE	Model 4-way IE
Central-Eastern Europe				-0.10*	-0.12*	-0.09*
				[-0.18,-0.02]	[-0.24,-0.01]	[-0.17,-0.01]
Other				-0.11**	-0.05	-0.11**
				[-0.18,-0.03]	[-0.19,0.08]	[-0.19,-0.04]
Test score	0.44***	0.44***	0.44***	0.44***	0.44***	0.44***
	[0.41,0.47]	[0.41,0.47]	[0.41,0.47]	[0.41,0.47]	[0.41,0.47]	[0.41,0.47]
Reasoning score	0.12***	0.12***	0.12***	0.12***	0.12***	0.12***
	[0.09,0.15]	[0.09,0.14]	[0.09,0.15]	[0.09,0.15]	[0.09,0.14]	[0.09,0.14]
Perceptual speed score	0.07***	0.07***	0.07***	0.07***	0.07***	0.07***
	[0.05,0.09]	[0.05,0.09]	[0.05,0.09]	[0.05,0.09]	[0.05,0.09]	[0.05,0.09]
School type (ref: *Hauptschule*)						
SmmB	-0.13**	-0.13**	-0.13**	-0.13**	-0.13**	-0.13**
	[-0.21,-0.05]	[-0.21,-0.05]	[-0.21,-0.05]	[-0.21,-0.05]	[-0.21,-0.05]	[-0.21,-0.05]
Realschule	-0.32***	-0.31***	-0.31***	-0.32***	-0.31***	-0.31***
	[-0.40,-0.23]	[-0.40,-0.23]	[-0.40,-0.23]	[-0.40,-0.23]	[-0.40,-0.23]	[-0.40,-0.23]
Gymnasium	-0.60***	-0.60***	-0.60***	-0.59***	-0.59***	-0.60***
	[-0.68,-0.51]	[-0.68,-0.51]	[-0.68,-0.51]	[-0.68,-0.51]	[-0.68,-0.51]	[-0.68,-0.51]

Continued on the next page

Table S8: Continuation from the previous page

	Model no IE	Model 2-way IE	Model 4-way IE	Model no IE	Model 2-way IE	Model 4-way IE
Interactions						
Minority x overweight/obese		0.03				
		[-0.10,0.17]				
Minority x girl		-0.07				
		[-0.16,0.01]				
Minority x SES (z)		-0.00				
		[-0.05,0.04]				
Overweight/obese x girl		-0.01			-0.01	
		[-0.14,0.11]			[-0.14,0.11]	
Overweight/obese x SES (z)		-0.02			-0.02	
		[-0.08,0.05]			[-0.08,0.05]	
Girl x SES (z)		0.07**			0.07**	
		[0.03,0.11]			[0.03,0.11]	
Majority x non-overweight/obese x girl x SES (z)			0.09***			
			[0.04,0.15]			
Majority x overweight/obese x boy x SES (z)			-0.03			
			[-0.12,0.07]			

Continued on the next page

Table S8: Continuation from the previous page

	Model no IE	Model 2-way IE	Model 4-way IE	Model no IE	Model 2-way IE	Model 4-way IE
Majority x overweight/obese x girl x SES (z)			0.11			
			[-0.02,0.24]			
Minority x non-overweight/obese x boy x SES (z)			0.03			
			[-0.04,0.11]			
Minority x non-overweight/obese x girl x SES (z)			0.06			
			[-0.01,0.13]			
Minority x overweight/obese x boy x SES (z)			-0.00			
			[-0.13,0.13]			
Minority x overweight/obese x girl x SES (z)			0.05			
			[-0.13,0.23]			
Turkey x overweight/obese					-0.12	
					[-0.37,0.13]	
FSU x overweight/obese					0.09	
					[-0.15,0.32]	
NW+South Europe x overweight/obese					0.22	
					[-0.13,0.56]	
Central-Eastern Europe x overweight/obese					-0.02	
					[-0.26,0.22]	

Continued on the next page

Table S8: Continuation from the previous page

	Model no IE	Model 2-way IE	Model 4-way IE	Model no IE	Model 2-way IE	Model 4-way IE
Other x overweight/obese					0.11	
					[-0.11,0.34]	
Turkey x girl					-0.03	
					[-0.20,0.14]	
FSU x girl					-0.22*	
					[-0.40,-0.04]	
N NW+South Europe x girl					-0.02	
					[-0.22,0.18]	
Central-Eastern Europe x girl					0.06	
					[-0.08,0.20]	
Other x girl					-0.15	
					[-0.33,0.03]	
Turkey x SES (z)					-0.04	
					[-0.15,0.07]	
FSU x SES (z)					0.01	
					[-0.09,0.10]	
NW+South Europe x SES (z)					0.04	
					[-0.06,0.13]	

Continued on the next page

Table S8: Continuation from the previous page

	Model no IE	Model 2-way IE	Model 4-way IE	Model no IE	Model 2-way IE	Model 4-way IE
Central-Eastern Europe x SES (z)					0.03	
					[-0.06,0.12]	
Other x SES (z)					-0.01	
					[-0.08,0.07]	
Majority x non-overweight/obese x girl x SES (z)						0.09***
						[0.04,0.14]
Majority x overweight/obese x boy x SES (z)						-0.02
						[-0.12,0.07]
Majority x overweight/obese x girl x SES (z)						0.11
						[-0.02,0.24]
Turkey x non-overweight/obese x boy x SES (z)						0.03
						[-0.12,0.19]
Turkey x non-overweight/obese x girl x SES (z)						-0.03
						[-0.18,0.12]
Turkey x overweight/obese x boy x SES (z)						-0.01
						[-0.26,0.23]
Turkey x overweight/obese x girl x SES (z)						0.06
						[-0.30,0.43]

Continued on the next page

Table S8: Continuation from the previous page

	Model no IE	Model 2-way IE	Model 4-way IE	Model no IE	Model 2-way IE	Model 4-way IE
FSU x non-overweight/obese x boy x SES (z)						-0.03
						[-0.18,0.13]
FSU x non-overweight/obese x girl x SES (z)						0.13*
						[0.00,0.26]
FSU x overweight/obese x boy x SES (z)						0.02
						[-0.26,0.29]
FSU x overweight/obese x girl x SES (z)						-0.38
						[-1.09,0.34]
NW+South Europe x non-overweight/obese x boy x SES (z)						0.03
						[-0.14,0.20]
NW+South Europe x non-overweight/obese x girl x SES (z)						0.10
						[-0.03,0.24]
NW+South Europe x overweight/obese x boy x SES (z)						0.02
						[-0.38,0.42]
NW+South Europe x overweight/obese x girl x SES (z)						0.25
						[-0.25,0.76]
Central-Eastern Europe x non-overweight/obese x boy x SES (z)						0.14*
						[0.02,0.27]

Continued on the next page

Table S8: Continuation from the previous page

	Model no IE	Model 2-way IE	Model 4-way IE	Model no IE	Model 2-way IE	Model 4-way IE
Central-Eastern Europe x non-overweight/obese x girl x SES (z)						0.03
						[-0.10,0.17]
Central-Eastern Europe x overweight/obese x boy x SES (z)						0.01
						[-0.27,0.30]
Central-Eastern Europe x overweight/obese x girl x SES (z)						0.16
						[-0.19,0.51]
Other x non-overweight/obese x boy x SES (z)						-0.00
						[-0.13,0.13]
Other x non-overweight/obese x girl x SES (z)						0.07
						[-0.05,0.19]
Other x overweight/obese x boy x SES (z)						-0.02
						[-0.26,0.22]
Other x overweight/obese x girl x SES (z)						0.02
						[-0.22,0.27]
Intercept	0.29***	0.28***	0.29***	0.29***	0.28***	0.29***
	[0.23,0.36]	[0.22,0.35]	[0.23,0.36]	[0.23,0.35]	[0.21,0.34]	[0.23,0.36]
SD(school)	0.17***	0.17***	0.17***	0.17***	0.17***	0.17***
	[0.13,0.22]	[0.13,0.22]	[0.13,0.22]	[0.13,0.22]	[0.13,0.22]	[0.13,0.22]

Continued on the next page

Table S8: Continuation from the previous page

	Model no IE	Model 2-way IE	Model 4-way IE	Model no IE	Model 2-way IE	Model 4-way IE
SD(class)	0.23***	0.23***	0.23***	0.23***	0.23***	0.22***
	[0.20,0.27]	[0.19,0.27]	[0.19,0.26]	[0.19,0.26]	[0.19,0.26]	[0.19,0.26]
Sigma	0.87***	0.87***	0.87***	0.87***	0.87***	0.87***
	[0.86,0.89]	[0.86,0.89]	[0.86,0.89]	[0.86,0.89]	[0.85,0.89]	[0.85,0.89]
N	13964	13964	13964	13964	13964	13964

Note: ***p≤0.001, **p≤0.01, *p≤0.05

Source: NEPS SC4 (based on m = 50 multiple imputed datasets); weighted data, our own calculations.
