# Supplementary material for: Does chubby Can get lower grades than skinny Sophie? Using an intersectional approach to uncover grading bias in German secondary schools
Source: PLoS One. 2024 Jul 3;19(7):e0305703. doi: 10.1371/journal.pone.0305703 (PMC11221685; doi:10.1371/journal.pone.0305703)
Supplement: S9 Table — (PDF) [file pone.0305703.s018.pdf]

Table S9: Multilevel-linear regression results (regression coefficients and [95% confidence intervals]) predicting school Grades in Physics (Intersectional models).

	Model no IE	Model 2-way IE	Model 4-way IE	Model no IE	Model 2-way IE	Model 4-way IE
Gender (ref: boy)						
Girl	-0.10***	-0.10***	-0.10***	-0.10***	-0.10***	-0.10***
	[-0.15,-0.06]	[-0.15,-0.05]	[-0.15,-0.06]	[-0.15,-0.06]	[-0.15,-0.05]	[-0.15,-0.06]
BMI (ref: non-overweight/obese)						
Overweight/obese	-0.08*	-0.11*	-0.08*	-0.08*	-0.11*	-0.08*
	[-0.15,-0.02]	[-0.20,-0.02]	[-0.15,-0.01]	[-0.15,-0.02]	[-0.20,-0.02]	[-0.14,-0.01]
SES (z)	0.05***	0.06**	0.07**	0.06***	0.06**	0.07**
	[0.03,0.08]	[0.02,0.10]	[0.02,0.11]	[0.03,0.08]	[0.02,0.10]	[0.02,0.11]
Minority status / group (ref: majority)						
Minority	-0.08**	-0.08*	-0.08**			
	[-0.13,-0.03]	[-0.16,-0.01]	[-0.14,-0.03]			
Turkey				-0.04	-0.12	-0.11
				[-0.14,0.06]	[-0.31,0.06]	[-0.23,0.01]
FSU				-0.03	0.00	-0.02
				[-0.13,0.07]	[-0.16,0.16]	[-0.13,0.09]
NW+South Europe				-0.13*	-0.19	-0.13*
				[-0.24,-0.02]	[-0.37,0.00]	[-0.24,-0.01]

Continued on the next page

Table S9: Continuation from the previous page

	Model no IE	Model 2-way IE	Model 4-way IE	Model no IE	Model 2-way IE	Model 4-way IE
Central-Eastern Europe				-0.05	-0.08	-0.06
				[-0.15,0.04]	[-0.22,0.06]	[-0.15,0.04]
Other				-0.12**	-0.09	-0.13**
				[-0.20,-0.05]	[-0.20,0.02]	[-0.21,-0.05]
Test score	0.23***	0.23***	0.23***	0.23***	0.23***	0.23***
	[0.20,0.25]	[0.20,0.25]	[0.20,0.25]	[0.20,0.26]	[0.20,0.26]	[0.20,0.26]
Reasoning score	0.08***	0.08***	0.08***	0.08***	0.08***	0.08***
	[0.06,0.10]	[0.06,0.10]	[0.06,0.10]	[0.06,0.11]	[0.06,0.11]	[0.06,0.10]
Perceptual speed score	0.07***	0.07***	0.07***	0.07***	0.07***	0.07***
	[0.04,0.09]	[0.04,0.09]	[0.04,0.09]	[0.04,0.09]	[0.04,0.09]	[0.04,0.09]
School type (ref: *Hauptschule*)						
SmmB	-0.17**	-0.17**	-0.17**	-0.17**	-0.17**	-0.17**
	[-0.28,-0.07]	[-0.27,-0.06]	[-0.27,-0.06]	[-0.28,-0.06]	[-0.27,-0.06]	[-0.27,-0.06]
Realschule	-0.28***	-0.28***	-0.28***	-0.28***	-0.28***	-0.28***
	[-0.37,-0.18]	[-0.37,-0.18]	[-0.37,-0.18]	[-0.37,-0.18]	[-0.37,-0.18]	[-0.37,-0.18]
Gymnasium	-0.26***	-0.26***	-0.26***	-0.26***	-0.26***	-0.26***
	[-0.36,-0.16]	[-0.36,-0.16]	[-0.36,-0.16]	[-0.36,-0.16]	[-0.36,-0.16]	[-0.36,-0.16]

Continued on the next page

Table S9: Continuation from the previous page

	Model no IE	Model 2-way IE	Model 4-way IE	Model no IE	Model 2-way IE	Model 4-way IE
Interactions						
Minority x overweight/obese		0.06				
		[-0.08,0.20]				
Minority x girl		-0.02				
		[-0.11,0.07]				
Minority x SES (z)		-0.04				
		[-0.09,0.02]				
Overweight/obese x girl		0.02			0.03	
		[-0.12,0.17]			[-0.12,0.18]	
Overweight/obese x SES (z)		-0.00			-0.00	
		[-0.08,0.07]			[-0.08,0.07]	
Girl x SES (z)		0.01			0.01	
		[-0.04,0.05]			[-0.04,0.05]	
Majority x non-overweight/obese x girl x SES (z)			0.00			
			[-0.05,0.06]			
Majority x overweight/obese x boy x SES (z)			-0.04			
			[-0.14,0.06]			

Continued on the next page

Table S9: Continuation from the previous page

	Model no IE	Model 2-way IE	Model 4-way IE	Model no IE	Model 2-way IE	Model 4-way IE
Majority x overweight/obese x girl x SES (z)			0.05			
			[-0.10,0.20]			
Minority x non-overweight/obese x boy x SES (z)			-0.04			
			[-0.11,0.04]			
Minority x non-overweight/obese x girl x SES (z)			-0.05			
			[-0.12,0.03]			
Minority x overweight/obese x boy x SES (z)			-0.04			
			[-0.20,0.13]			
Minority x overweight/obese x girl x SES (z)			-0.00			
			[-0.19,0.18]			
Turkey x overweight/obese					-0.03	
					[-0.31,0.24]	
FSU x overweight/obese					0.04	
					[-0.30,0.37]	
NW+South Europe x overweight/obese					0.28	
					[-0.08,0.64]	
Central-Eastern Europe x overweight/obese					0.15	
					[-0.09,0.40]	

Continued on the next page

Table S9: Continuation from the previous page

	Model no IE	Model 2-way IE	Model 4-way IE	Model no IE	Model 2-way IE	Model 4-way IE
Other x overweight/obese					-0.00	
					[-0.22,0.21]	
Turkey x girl					0.03	
					[-0.17,0.24]	
FSU x girl					-0.05	
					[-0.24,0.14]	
NW+South Europe x girl					0.03	
					[-0.19,0.25]	
Central-Eastern Europe x girl					0.00	
					[-0.17,0.17]	
Other x girl					-0.07	
					[-0.21,0.08]	
Turkey x SES (z)					-0.12*	
					[-0.24,-0.00]	
FSU x SES (z)					0.01	
					[-0.11,0.14]	
NW+South Europe x SES (z)					0.03	
					[-0.07,0.13]	

Continued on the next page

Table S9: Continuation from the previous page

	Model no IE	Model 2-way IE	Model 4-way IE	Model no IE	Model 2-way IE	Model 4-way IE
Central-Eastern Europe x SES (z)					-0.02	
					[-0.12,0.07]	
Other x SES (z)					-0.04	
					[-0.12,0.04]	
Majority x non-overweight/obese x girl x SES (z)						0.00
						[-0.05,0.06]
Majority x overweight/obese x boy x SES (z)						-0.04
						[-0.14,0.06]
Majority x overweight/obese x girl x SES (z)						0.05
						[-0.10,0.20]
Turkey x non-overweight/obese x boy x SES (z)						-0.07
						[-0.22,0.09]
Turkey x non-overweight/obese x girl x SES (z)						-0.21*
						[-0.40,-0.03]
Turkey x overweight/obese x boy x SES (z)						-0.04
						[-0.29,0.22]
Turkey x overweight/obese x girl x SES (z)						-0.07
						[-0.53,0.39]

Continued on the next page

Table S9: Continuation from the previous page

	Model no IE	Model 2-way IE	Model 4-way IE	Model no IE	Model 2-way IE	Model 4-way IE
FSU x non-overweight/obese x boy x SES (z)						-0.03
						[-0.20,0.15]
FSU x non-overweight/obese x girl x SES (z)						0.04
						[-0.12,0.21]
FSU x overweight/obese x boy x SES (z)						-0.08
						[-0.38,0.21]
FSU x overweight/obese x girl x SES (z)						0.43
						[-0.21,1.07]
NW+South Europe x non-overweight/obese x boy x SES (z)						-0.02
						[-0.18,0.15]
NW+South Europe x non-overweight/obese x girl x SES (z)						0.02
						[-0.13,0.18]
NW+South Europe x overweight/obese x boy x SES (z)						0.08
						[-0.36,0.52]
NW+South Europe x overweight/obese x girl x SES (z)						0.06
						[-0.51,0.64]
Central-Eastern Europe x non-overweight/obese x boy x SES (z)						0.03
						[-0.12,0.18]

Continued on the next page

Table S9: Continuation from the previous page

	Model no IE	Model 2-way IE	Model 4-way IE	Model no IE	Model 2-way IE	Model 4-way IE
Central-Eastern Europe x non-overweight/obese x girl x SES (z)						-0.04
						[-0.17,0.10]
Central-Eastern Europe x overweight/obese x boy x SES (z)						-0.09
						[-0.38,0.20]
Central-Eastern Europe x overweight/obese x girl x SES (z)						-0.18
						[-0.65,0.29]
Other x non-overweight/obese x boy x SES (z)						-0.07
						[-0.20,0.06]
Other x non-overweight/obese x girl x SES (z)						-0.03
						[-0.15,0.09]
Other x overweight/obese x boy x SES (z)						-0.03
						[-0.38,0.33]
Other x overweight/obese x girl x SES (z)						-0.01
						[-0.22,0.20]
Intercept	0.26***	0.26***	0.25***	0.26***	0.26***	0.25***
	[0.19,0.33]	[0.19,0.33]	[0.18,0.33]	[0.19,0.33]	[0.19,0.33]	[0.18,0.32]
SD(school)	0.27***	0.27***	0.27***	0.27***	0.27***	0.27***
	[0.24,0.30]	[0.24,0.30]	[0.24,0.30]	[0.24,0.30]	[0.24,0.30]	[0.24,0.30]

Continued on the next page

Table S9: Continuation from the previous page

	Model no IE	Model 2-way IE	Model 4-way IE	Model no IE	Model 2-way IE	Model 4-way IE
SD(class)	0.20***	0.20***	0.20***	0.20***	0.20***	0.20***
	[0.17,0.24]	[0.17,0.24]	[0.17,0.24]	[0.17,0.24]	[0.17,0.24]	[0.17,0.24]
Sigma	0.89***	0.89***	0.89***	0.89***	0.89***	0.89***
	[0.88,0.91]	[0.88,0.91]	[0.88,0.91]	[0.88,0.91]	[0.88,0.91]	[0.88,0.91]
N	12956	12956	12956	12956	12956	12956

Note: ***p≤0.001, **p≤0.01, *p≤0.05

Source: NEPS SC4 (based on m = 50 multiple imputed datasets); weighted data, our own calculations.
