# Supplementary material for: Does chubby Can get lower grades than skinny Sophie? Using an intersectional approach to uncover grading bias in German secondary schools
Source: PLoS One. 2024 Jul 3;19(7):e0305703. doi: 10.1371/journal.pone.0305703 (PMC11221685; doi:10.1371/journal.pone.0305703)
Supplement: S10 Table — (PDF) [file pone.0305703.s019.pdf]

Table S10: Multilevel-linear regression results (regression coefficients and [95% confidence intervals]) predicting school Grades in Chemistry (Intersectional models).

	Model no IE	Model 2-way IE	Model 4-way IE	Model no IE	Model 2-way IE	Model 4-way IE
Gender (ref: boy)						
Girl	0.01	-0.01	0.01	0.01	-0.01	0.01
	[-0.04,0.06]	[-0.07,0.05]	[-0.04,0.06]	[-0.04,0.06]	[-0.07,0.05]	[-0.04,0.06]
BMI (ref: non-overweight/obese)						
Overweight/obese	-0.07*	-0.08	-0.08*	-0.07*	-0.08	-0.08
	[-0.15,-0.00]	[-0.19,0.02]	[-0.16,-0.00]	[-0.15,-0.00]	[-0.19,0.02]	[-0.15,0.00]
SES (z)	0.04**	0.02	0.03	0.04***	0.02	0.03
	[0.02,0.07]	[-0.01,0.06]	[-0.01,0.06]	[0.02,0.07]	[-0.01,0.06]	[-0.01,0.06]
Minority status / group (ref: majority)						
Minority	-0.06*	-0.10*	-0.06*			
	[-0.12,-0.01]	[-0.18,-0.02]	[-0.11,-0.01]			
Turkey				-0.02	-0.02	-0.04
				[-0.11,0.08]	[-0.18,0.14]	[-0.17,0.08]
FSU				-0.03	-0.01	-0.02
				[-0.13,0.07]	[-0.16,0.14]	[-0.13,0.08]
NW+South Europe				-0.11*	-0.19*	-0.12*
				[-0.21,-0.01]	[-0.37,-0.01]	[-0.22,-0.02]

Continued on the next page

Table S10: Continuation from the previous page

	Model no IE	Model 2-way IE	Model 4-way IE	Model no IE	Model 2-way IE	Model 4-way IE
Central-Eastern Europe				-0.10*	-0.20*	-0.09
				[-0.19,-0.00]	[-0.35,-0.04]	[-0.18,0.00]
Other				-0.05	-0.09	-0.06
				[-0.13,0.03]	[-0.21,0.03]	[-0.14,0.02]
Test score	0.25***	0.24***	0.24***	0.25***	0.25***	0.25***
	[0.22,0.27]	[0.22,0.27]	[0.22,0.27]	[0.22,0.27]	[0.22,0.27]	[0.22,0.27]
Reasoning score	0.07***	0.07***	0.07***	0.07***	0.07***	0.07***
	[0.04,0.10]	[0.04,0.10]	[0.04,0.10]	[0.04,0.10]	[0.04,0.10]	[0.04,0.10]
Perceptual speed score	0.07***	0.07***	0.07***	0.07***	0.07***	0.07***
	[0.04,0.09]	[0.04,0.09]	[0.04,0.09]	[0.04,0.09]	[0.04,0.09]	[0.04,0.09]
School type (ref: *Hauptschule*)						
SmmB	-0.15**	-0.15**	-0.15**	-0.15**	-0.15**	-0.15**
	[-0.25,-0.05]	[-0.25,-0.05]	[-0.25,-0.05]	[-0.25,-0.05]	[-0.25,-0.06]	[-0.25,-0.05]
Realschule	-0.23***	-0.23***	-0.23***	-0.23***	-0.23***	-0.23***
	[-0.33,-0.13]	[-0.33,-0.13]	[-0.33,-0.13]	[-0.33,-0.13]	[-0.33,-0.13]	[-0.33,-0.13]
Gymnasium	-0.27***	-0.27***	-0.27***	-0.27***	-0.27***	-0.27***
	[-0.37,-0.17]	[-0.37,-0.17]	[-0.37,-0.17]	[-0.37,-0.17]	[-0.37,-0.17]	[-0.37,-0.17]

Continued on the next page

Table S10: Continuation from the previous page

	Model no IE	Model 2-way IE	Model 4-way IE	Model no IE	Model 2-way IE	Model 4-way IE
Interactions						
Minority x overweight/obese		-0.00				
		[-0.16,0.16]				
Minority x girl		0.08				
		[-0.01,0.17]				
Minority x SES (z)		0.02				
		[-0.04,0.07]				
Overweight/obese x girl		0.02			0.01	
		[-0.13,0.16]			[-0.14,0.15]	
Overweight/obese x SES (z)		-0.03			-0.04	
		[-0.11,0.05]			[-0.11,0.04]	
Girl x SES (z)		0.04			0.03	
		[-0.01,0.08]			[-0.01,0.08]	
Majority x non-overweight/obese x girl x SES (z)			0.03			
			[-0.02,0.08]			
Majority x overweight/obese x boy x SES (z)			-0.05			
			[-0.15,0.05]			

Continued on the next page

Table S10: Continuation from the previous page

	Model no IE	Model 2-way IE	Model 4-way IE	Model no IE	Model 2-way IE	Model 4-way IE
Majority x overweight/obese x girl x SES (z)			0.04			
			[-0.12,0.20]			
Minority x non-overweight/obese x boy x SES (z)			0.02			
			[-0.06,0.10]			
Minority x non-overweight/obese x girl x SES (z)			0.04			
			[-0.03,0.11]			
Minority x overweight/obese x boy x SES (z)			-0.02			
			[-0.18,0.14]			
Minority x overweight/obese x girl x SES (z)			0.06			
			[-0.15,0.27]			
Turkey x overweight/obese					-0.18	
					[-0.48,0.13]	
FSU x overweight/obese					-0.11	
					[-0.48,0.25]	
NW+South Europe x overweight/obese					0.16	
					[-0.22,0.54]	
Central-Eastern Europe x overweight/obese					-0.00	
					[-0.26,0.25]	

Continued on the next page

Table S10: Continuation from the previous page

	Model no IE	Model 2-way IE	Model 4-way IE	Model no IE	Model 2-way IE	Model 4-way IE
Other x overweight/obese					0.11	
					[-0.13,0.34]	
Turkey x girl					0.02	
					[-0.17,0.21]	
FSU x girl					-0.01	
					[-0.21,0.19]	
NW+South Europe x girl					0.09	
					[-0.12,0.30]	
Central-Eastern Europe x girl					0.21*	
					[0.03,0.40]	
Other x girl					0.04	
					[-0.11,0.18]	
Turkey x SES (z)					-0.03	
					[-0.16,0.11]	
FSU x SES (z)					0.01	
					[-0.09,0.12]	
NW+South Europe x SES (z)					0.11	
					[-0.00,0.22]	

Continued on the next page

Table S10: Continuation from the previous page

	Model no IE	Model 2-way IE	Model 4-way IE	Model no IE	Model 2-way IE	Model 4-way IE
Central-Eastern Europe x SES (z)					0.07	
					[-0.04,0.18]	
Other x SES (z)					-0.02	
					[-0.10,0.06]	
Majority x non-overweight/obese x girl x SES (z)						0.03
						[-0.02,0.08]
Majority x overweight/obese x boy x SES (z)						-0.05
						[-0.15,0.05]
Majority x overweight/obese x girl x SES (z)						0.04
						[-0.12,0.20]
Turkey x non-overweight/obese x boy x SES (z)						0.01
						[-0.15,0.18]
Turkey x non-overweight/obese x girl x SES (z)						-0.07
						[-0.24,0.11]
Turkey x overweight/obese x boy x SES (z)						0.02
						[-0.26,0.31]
Turkey x overweight/obese x girl x SES (z)						0.03
						[-0.37,0.44]

Continued on the next page

Table S10: Continuation from the previous page

	Model no IE	Model 2-way IE	Model 4-way IE	Model no IE	Model 2-way IE	Model 4-way IE
FSU x non-overweight/obese x boy x SES (z)						-0.01
						[-0.16,0.14]
FSU x non-overweight/obese x girl x SES (z)						0.05
						[-0.09,0.19]
FSU x overweight/obese x boy x SES (z)						0.01
						[-0.26,0.29]
FSU x overweight/obese x girl x SES (z)						0.34
						[-0.26,0.94]
NW+South Europe x non-overweight/obese x boy x SES (z)						0.04
						[-0.15,0.22]
NW+South Europe x non-overweight/obese x girl x SES (z)						0.17**
						[0.05,0.29]
NW+South Europe x overweight/obese x boy x SES (z)						0.10
						[-0.32,0.52]
NW+South Europe x overweight/obese x girl x SES (z)						0.20
						[-0.51,0.91]
Central-Eastern Europe x non-overweight/obese x boy x SES (z)						0.12
						[-0.06,0.30]

Continued on the next page

Table S10: Continuation from the previous page

	Model no IE	Model 2-way IE	Model 4-way IE	Model no IE	Model 2-way IE	Model 4-way IE
Central-Eastern Europe x non-overweight/obese x girl x SES (z)						0.08
						[-0.05,0.21]
Central-Eastern Europe x overweight/obese x boy x SES (z)						0.02
						[-0.25,0.29]
Central-Eastern Europe x overweight/obese x girl x SES (z)						-0.10
						[-0.46,0.27]
Other x non-overweight/obese x boy x SES (z)						-0.02
						[-0.15,0.11]
Other x non-overweight/obese x girl x SES (z)						0.01
						[-0.10,0.12]
Other x overweight/obese x boy x SES (z)						-0.15
						[-0.47,0.16]
Other x overweight/obese x girl x SES (z)						0.05
						[-0.29,0.39]
Intercept	0.17***	0.19***	0.17***	0.17***	0.19***	0.17***
	[0.10,0.25]	[0.11,0.26]	[0.10,0.25]	[0.10,0.25]	[0.11,0.26]	[0.10,0.25]
SD(school)	0.26***	0.26***	0.26***	0.26***	0.26***	0.26***
	[0.22,0.30]	[0.22,0.30]	[0.22,0.30]	[0.22,0.30]	[0.22,0.30]	[0.22,0.30]

Continued on the next page

Table S10: Continuation from the previous page

	Model no IE	Model 2-way IE	Model 4-way IE	Model no IE	Model 2-way IE	Model 4-way IE
SD(class)	0.22***	0.22***	0.22***	0.22***	0.22***	0.22***
	[0.19,0.26]	[0.19,0.26]	[0.19,0.26]	[0.19,0.26]	[0.19,0.26]	[0.19,0.26]
Sigma	0.89***	0.89***	0.89***	0.89***	0.89***	0.89***
	[0.88,0.91]	[0.88,0.91]	[0.88,0.91]	[0.88,0.91]	[0.88,0.91]	[0.88,0.91]
N	12898	12898	12898	12898	12898	12898

Note: ***p≤0.001, **p≤0.01, *p≤0.05

Source: NEPS SC4 (based on m = 50 multiple imputed datasets); weighted data, our own calculations.
