# Supplementary material for: Does chubby Can get lower grades than skinny Sophie? Using an intersectional approach to uncover grading bias in German secondary schools
Source: PLoS One. 2024 Jul 3;19(7):e0305703. doi: 10.1371/journal.pone.0305703 (PMC11221685; doi:10.1371/journal.pone.0305703)
Supplement: S11 Table — (PDF) [file pone.0305703.s020.pdf]

Table S11: Multilevel-linear regression results (regression coefficients and [95% confidence intervals]) predicting school Grades in Biology (Intersectional models).

	Model no IE	Model 2-way IE	Model 4-way IE	Model no IE	Model 2-way IE	Model 4-way IE
Gender (ref: boy)						
Girl	0.20***	0.21***	0.21***	0.20***	0.21***	0.21***
	[0.16,0.25]	[0.16,0.27]	[0.16,0.25]	[0.16,0.25]	[0.16,0.27]	[0.16,0.25]
BMI (ref: non-overweight/obese)						
Overweight/obese	-0.07	-0.11*	-0.07	-0.07	-0.11*	-0.07
	[-0.15,0.00]	[-0.20,-0.02]	[-0.15,0.00]	[-0.15,0.00]	[-0.20,-0.02]	[-0.15,0.00]
SES (z)	0.06***	0.05**	0.05*	0.06***	0.05**	0.05*
	[0.04,0.09]	[0.01,0.09]	[0.01,0.09]	[0.04,0.09]	[0.02,0.09]	[0.01,0.09]
Minority status / group (ref: majority)						
Minority	-0.03	-0.03	-0.03			
	[-0.08,0.02]	[-0.11,0.05]	[-0.08,0.02]			
Turkey				-0.02	-0.02	-0.07
				[-0.12,0.08]	[-0.20,0.16]	[-0.21,0.06]
FSU				-0.03	-0.02	-0.03
				[-0.13,0.07]	[-0.18,0.15]	[-0.14,0.08]
NW+South Europe				-0.06	-0.07	-0.06
				[-0.19,0.07]	[-0.28,0.15]	[-0.19,0.06]

Continued on the next page

Table S11: Continuation from the previous page

	Model no IE	Model 2-way IE	Model 4-way IE	Model no IE	Model 2-way IE	Model 4-way IE
Central-Eastern Europe				-0.00	-0.05	0.00
				[-0.09,0.09]	[-0.19,0.10]	[-0.09,0.09]
Other				-0.04	-0.03	-0.05
				[-0.13,0.05]	[-0.16,0.10]	[-0.14,0.03]
Test score	0.23***	0.23***	0.23***	0.23***	0.23***	0.23***
	[0.20,0.26]	[0.20,0.26]	[0.20,0.26]	[0.20,0.26]	[0.20,0.26]	[0.20,0.26]
Reasoning score	0.05***	0.05***	0.05***	0.05***	0.05***	0.05***
	[0.02,0.07]	[0.02,0.07]	[0.02,0.07]	[0.02,0.07]	[0.02,0.07]	[0.02,0.07]
Perceptual speed score	0.05***	0.05***	0.05***	0.05***	0.05***	0.05***
	[0.02,0.08]	[0.02,0.08]	[0.02,0.08]	[0.02,0.08]	[0.03,0.08]	[0.02,0.08]
School type (ref: *Hauptschule*)						
SmmB	-0.06	-0.06	-0.06	-0.06	-0.06	-0.06
	[-0.16,0.05]	[-0.16,0.05]	[-0.16,0.05]	[-0.16,0.04]	[-0.17,0.04]	[-0.17,0.04]
Realschule	-0.10*	-0.10	-0.10	-0.10*	-0.10*	-0.11*
	[-0.21,-0.00]	[-0.21,0.00]	[-0.21,0.00]	[-0.21,-0.00]	[-0.21,-0.00]	[-0.21,-0.00]
Gymnasium	-0.11*	-0.11*	-0.11*	-0.11*	-0.11*	-0.11*
	[-0.21,-0.01]	[-0.21,-0.01]	[-0.21,-0.01]	[-0.21,-0.01]	[-0.21,-0.01]	[-0.21,-0.01]

Continued on the next page

Table S11: Continuation from the previous page

	Model no IE	Model 2-way IE	Model 4-way IE	Model no IE	Model 2-way IE	Model 4-way IE
Interactions						
Minority x overweight/obese		0.10				
		[-0.06,0.25]				
Minority x girl		-0.03				
		[-0.13,0.07]				
Minority x SES (z)		-0.01				
		[-0.06,0.04]				
Overweight/obese x girl		0.03			0.03	
		[-0.10,0.17]			[-0.10,0.16]	
Overweight/obese x SES (z)		0.02			0.01	
		[-0.06,0.09]			[-0.07,0.09]	
Girl x SES (z)		0.03			0.03	
		[-0.01,0.08]			[-0.01,0.08]	
Majority x non-overweight/obese x girl x SES (z)			0.03			
			[-0.02,0.08]			
Majority x overweight/obese x boy x SES (z)			0.03			
			[-0.08,0.13]			

Continued on the next page

Table S11: Continuation from the previous page

	Model no IE	Model 2-way IE	Model 4-way IE	Model no IE	Model 2-way IE	Model 4-way IE
Majority x overweight/obese x girl x SES (z)			0.05			
			[-0.09,0.18]			
Minority x non-overweight/obese x boy x SES (z)			-0.02			
			[-0.10,0.07]			
Minority x non-overweight/obese x girl x SES (z)			0.03			
			[-0.04,0.09]			
Minority x overweight/obese x boy x SES (z)			-0.03			
			[-0.22,0.15]			
Minority x overweight/obese x girl x SES (z)			0.02			
			[-0.17,0.21]			
Turkey x overweight/obese					-0.09	
					[-0.42,0.25]	
FSU x overweight/obese					-0.08	
					[-0.37,0.22]	
NW+South Europe x overweight/obese					0.35	
					[-0.02,0.72]	
Central-Eastern Europe x overweight/obese					0.18	
					[-0.06,0.42]	

Continued on the next page

Table S11: Continuation from the previous page

	Model no IE	Model 2-way IE	Model 4-way IE	Model no IE	Model 2-way IE	Model 4-way IE
Other x overweight/obese					0.13	
					[-0.11,0.36]	
Turkey x girl					-0.07	
					[-0.27,0.14]	
FSU x girl					-0.01	
					[-0.22,0.20]	
NW+South Europe x girl					-0.08	
					[-0.33,0.16]	
Central-Eastern Europe x girl					0.04	
					[-0.14,0.23]	
Other x girl					-0.07	
					[-0.21,0.08]	
Turkey x SES (z)					-0.08	
					[-0.22,0.06]	
FSU x SES (z)					-0.01	
					[-0.10,0.09]	
NW+South Europe x SES (z)					0.09	
					[-0.04,0.23]	

Continued on the next page

Table S11: Continuation from the previous page

	Model no IE	Model 2-way IE	Model 4-way IE	Model no IE	Model 2-way IE	Model 4-way IE
Central-Eastern Europe x SES (z)					0.00	
					[-0.09,0.10]	
Other x SES (z)					-0.04	
					[-0.12,0.04]	
Majority x non-overweight/obese x girl x SES (z)						0.03
						[-0.02,0.08]
Majority x overweight/obese x boy x SES (z)						0.03
						[-0.08,0.13]
Majority x overweight/obese x girl x SES (z)						0.05
						[-0.09,0.18]
Turkey x non-overweight/obese x boy x SES (z)						-0.06
						[-0.26,0.14]
Turkey x non-overweight/obese x girl x SES (z)						-0.08
						[-0.25,0.08]
Turkey x overweight/obese x boy x SES (z)						-0.09
						[-0.43,0.25]
Turkey x overweight/obese x girl x SES (z)						0.12
						[-0.30,0.54]

Continued on the next page

Table S11: Continuation from the previous page

	Model no IE	Model 2-way IE	Model 4-way IE	Model no IE	Model 2-way IE	Model 4-way IE
FSU x non-overweight/obese x boy x SES (z)						-0.03
						[-0.18,0.12]
FSU x non-overweight/obese x girl x SES (z)						0.04
						[-0.09,0.18]
FSU x overweight/obese x boy x SES (z)						0.05
						[-0.23,0.33]
FSU x overweight/obese x girl x SES (z)						-0.04
						[-0.47,0.40]
NW+South Europe x non-overweight/obese x boy x SES (z)						0.03
						[-0.20,0.25]
NW+South Europe x non-overweight/obese x girl x SES (z)						0.16
						[-0.02,0.34]
NW+South Europe x overweight/obese x boy x SES (z)						-0.02
						[-0.45,0.42]
NW+South Europe x overweight/obese x girl x SES (z)						0.30
						[-0.32,0.93]
Central-Eastern Europe x non-overweight/obese x boy x SES (z)						0.03
						[-0.11,0.17]

Continued on the next page

Table S11: Continuation from the previous page

	Model no IE	Model 2-way IE	Model 4-way IE	Model no IE	Model 2-way IE	Model 4-way IE
Central-Eastern Europe x non-overweight/obese x girl x SES (z)						0.01
						[-0.13,0.14]
Central-Eastern Europe x overweight/obese x boy x SES (z)						0.03
						[-0.24,0.30]
Central-Eastern Europe x overweight/obese x girl x SES (z)						0.05
						[-0.33,0.43]
Other x non-overweight/obese x boy x SES (z)						-0.05
						[-0.19,0.09]
Other x non-overweight/obese x girl x SES (z)						0.02
						[-0.09,0.13]
Other x overweight/obese x boy x SES (z)						-0.13
						[-0.48,0.22]
Other x overweight/obese x girl x SES (z)						-0.10
						[-0.37,0.18]
Intercept	-0.02	-0.02	-0.02	-0.02	-0.02	-0.02
	[-0.09,0.05]	[-0.09,0.05]	[-0.09,0.05]	[-0.09,0.05]	[-0.09,0.05]	[-0.09,0.05]
SD(school)	0.22***	0.21***	0.21***	0.22***	0.21***	0.21***
	[0.17,0.27]	[0.17,0.27]	[0.17,0.27]	[0.17,0.27]	[0.17,0.27]	[0.17,0.27]

Continued on the next page

Table S11: Continuation from the previous page

	Model no IE	Model 2-way IE	Model 4-way IE	Model no IE	Model 2-way IE	Model 4-way IE
SD(class)	0.28***	0.28***	0.28***	0.28***	0.28***	0.28***
	[0.23,0.33]	[0.23,0.33]	[0.23,0.33]	[0.23,0.33]	[0.23,0.33]	[0.23,0.33]
Sigma	0.89***	0.89***	0.89***	0.89***	0.89***	0.89***
	[0.87,0.91]	[0.87,0.91]	[0.87,0.91]	[0.87,0.91]	[0.87,0.91]	[0.87,0.91]
N	12207	12207	12207	12207	12207	12207

Note: ***p≤0.001, **p≤0.01, *p≤0.05

Source: NEPS SC4 (based on m = 50 multiple imputed datasets); weighted data, our own calculations.
