# Supplementary material for: Correction: Does chubby Can get lower grades than skinny Sophie? Using an intersectional approach to uncover grading bias in German secondary schools
Source: PLoS One. 2025 Mar 5;20(3):e0320170. doi: 10.1371/journal.pone.0320170 (PMC11882040; doi:10.1371/journal.pone.0320170)
Supplement: S1 File — (PDF) [file pone.0320170.s001.pdf]

PLOS ONE

RESEARCH ARTICLE

# Does chubby can get lower grades than skinny Sophie? Using an intersectional approach to uncover grading bias in German secondary schools

**Richard Nennstiel**[1☯]*, **Sandra Gilgen**[1,2☯]

**1** 1 Department of Sociology of Education, University of Bern, Bern, Switzerland, **2** Empirical and Normative Knowledge and Data Centre of the URPP Human Reproduction Reloaded – H2R, University of Zurich, Zurich, Switzerland

☯ These authors contributed equally to this work.
* richard.nennstiel@unibe.ch

Check for updates

OPEN ACCESS

**Citation:** Nennstiel R, Gilgen S (2024) Does chubby can get lower grades than skinny Sophie? Using an intersectional approach to uncover grading bias in German secondary schools. PLoS ONE 19(7): e0305703. https://doi.org/10.1371/journal.pone.0305703

**Editor:** Eyal Bar-Haim, Bar-Ilan University, ISRAEL

**Received:** December 14, 2023

**Accepted:** June 2, 2024

**Published:** July 3, 2024

**Copyright:** © 2024 Nennstiel, Gilgen. This is an open access article distributed under the terms of the Creative Commons Attribution License, which permits unrestricted use, distribution, and reproduction in any medium, provided the original author and source are credited.

**Data Availability Statement:** The NEPS data used in this study are owned by a third party and the authors do not have permission to share the data. Data are available from the LIfBi (fdz@lifbi.de) for researchers who meet the criteria for access to confidential data. This paper uses data from the National Educational Panel Study (NEPS; see Blossfeld & Roßbach, 2019). The NEPS is carried out by the Leibniz Institute for Educational Trajectories (LIfBi, Germany) in cooperation with a nationwide network. Data and documents are made available for academic research and are accessible to registered users who have signed a

## Abstract

We aim to uncover grading bias by gender, socio-economic status, ethnic/migration background as well as body weight in the German secondary school system. Following an intersectional approach, we test whether—controlling for ability—students receive different grades depending on (the specific combination of) ascriptive characteristics. Using data from the fourth starting cohort (SC4, 13.0.0, first survey in year 9 in 2010) of the National Educational Panel Study (NEPS) consisting of more than 14,000 ninth graders, we compute the predicted differences in grades for the different groups of students depending on whether they are a boy or a girl, whether they are obese/overweight or not, their socio-economic status (SES) and ethnic background. We rely on a grade equation approach, assuming that discrepancies between observed grades and achievement as measured in standardised tests are evidence of biased grading. We control for two different competence tests—the Domain General Cognitive Functions (DGCF) and a standardised domain-specific competence test—as objective measures of ability as well as secondary school track. Even after controlling for different personality and behavioural traits—the "big five", the Strengths and Difficulties Questionnaire (SDQ), the Sick, Control, One, Fat and Food (SCOFF), health satisfaction and class retention—substantial differentials in grading across almost all factors and subjects remain. To account for the fact that many students may face bias on multiple grounds, we then compare the differences in predicted grades for groups with overlapping (dis)advantaging characteristics (e.g. low SES overweight Turkish boy vs a high SES non-overweight majority girl), while controlling for the objective ability measures. Significant differentials in grades are found in almost all cases, with the largest effect sizes for the subject German. We also compute models including all 2-way or 4-way interactions between the four axes of inequality and find the main effects largely unchanged. On the whole our findings are indicative of widespread additive intersectional effects of gender, social and ethnic origin as well as body weight on grading bias.

data use agreement with the LIfBi (fdz@lifbi.de). This release is uniquely identified by a DOI. The sub-datasets used in this research are SC4_pTarget_R_13-0-0.dta, SC4_Weights_R_13-0-0.dta, SC4_CohortProfile_R_13-0-0.dta, SC4_pParent_R_13-0-0.dta, SC4_xTargetCompetencies_R_13-0-0.dta. NEPS Network. (2023). National Educational Panel Study, Scientific Use File of Starting Cohort Grade 9. Leibniz Institute for Educational Trajectories (LIfBi), Bamberg. https://doi.org/10.5157/NEPS:SC4:13.0.0 Blossfeld, H.-P. & Roßbach, H.-G. (Eds.). (2019). Education as a lifelong process: The German National Educational Panel Study (NEPS). Edition ZfE (2nd ed.). Springer VS. The dataset on the BMI classification, "bmiage: BMI-for-age charts, 2 to 20 years, LMS parameters and selected smoothed BMI (kilograms/meters squared) percentiles, by sex and age," May 30, 2000 version, is provided by the CDC National Center for Health Statistics. This dataset is available to the public at https://www.cdc.gov/growthcharts/percentile_data_files.htm.

**Funding:** The author(s) received no specific funding for this work.

**Competing interests:** The authors have declared that no competing interests exist.

## Introduction

When it comes to educational success, school grades are of great importance as they greatly structure educational trajectories by restricting and enabling passages to higher tracks [1]. Furthermore, school grades are relevant signals that influence school to work transitions [2, 3], access to fields of study [4, 5] as well as opportunities on the job market [6]. There is also evidence suggesting that, in line with a self-fulfilling prophecy mechanism, while being overestimated by teachers is beneficial for future outcomes, being underestimated has negative consequences for students [7–9]. In light of their pivotal role, school grades should be an unbiased reflection of students' capabilities and performance, and it is especially important that they are not a reflection of teachers' underestimation of students.

If we look at absolute grade differences between specific groups, we see that, e.g. boys have higher grades in maths and in other STEM subjects (science, technology, engineering, and mathematics), while girls have higher grades in languages [10, 11]. Also, minority students have lower grades than majority students [12, 13] and children from lower social classes receive lower grades than their more privileged peers [14]. Furthermore, there are differences between children of different body types, with disadvantages for overweight and obese children compared to their peers [15–17].

These grade differentials could (partly) reflect differences in performance. As we know from international studies, there are group differences in objective test scores: On average, girls achieve higher scores in language tests and lower scores in mathematics than boys. Furthermore, minority students as well as overweight or obese students achieve lower scores than majority students and students who are not overweight or obese [15, 17–23]. However, ample evidence across different contexts suggests that there is more to it and that grading bias by teachers plays a significant role.

In this paper, we thus aim to uncover grading bias—understood as grading differentials for students with the same achievement levels due to unequal treatment by teachers—in German secondary schools [13, 17]. Existing research suggests grading bias against lower-class youth [17, 24–31] and minority [12, 13, 25, 28, 30–38] as well as overweight or obese [17, 39–42] pupils. Furthermore, there is also evidence for grading bias by gender, benefiting boys or girls depending on the subject [9–12, 25, 27, 30, 35, 43–52]. The most plausible explanation for grading bias, for which there is supporting evidence, is that teachers' evaluations of students' abilities are affected by the stereotypical beliefs they hold about specific groups [29, 37, 53]. Furthermore, students' classroom behaviour has been found to have an effect on grades [11, 54, 55].

There are numerous studies that examine the extent of grading bias based on ascriptive characteristics (e.g. gender, social class, ethnic origin, or body weight) using a variety of approaches such as surveys or experimental data. The majority of this research on grading bias focuses on mathematics and language skills. For studies that examine grading bias across many subjects, see, for example, [30, 43, 47]. Significantly fewer studies explore intersectional inequalities [56] in grading bias [31], despite the growing exploration of intersectionality in educational research [57–61]. Additionally, since an individual can, for example, be male, overweight, be of a minority ethnic origin and come from a lower-class background, in reality the factors affected by grading bias may intersect and result in a disadvantage that may be cumulative across characteristics or even greater than the sum of the individual negative outcomes. However, existing studies that do examine grading bias from an intersectional point of view often focus on the intersection of two characteristics (e.g. gender and ethnicity, gender and weight, or gender and social class; [17, 31, 52]). Therefore, the aim of our paper is to contribute both to the research on grading bias as well as to the literature on intersectionality in

educational research by examining grading bias while simultaneously taking into account the four characteristics: gender, ethnic origin, class background, and weight status. We also include not only language skills and mathematics, but also biology, chemistry and physics in our analysis of grading bias.

Since many results on grading bias in Germany have been obtained through experiments in which teachers rated hypothetical students, the question remains to what extent these results are transferable to real-life classrooms. Some have argued that in real life, where teachers know their pupils and thus need not resort to stereotypes to assess their abilities, grading bias might not be that much of an issue [46, 62]. However, there is a lack of large-scale research on grading bias in Germany to back up this claim (for a recent exception, see [17]). Using the National Educational Panel Study (NEPS), a nationally representative large scale survey from Germany, we thus aim to uncover the extent to which grading bias (in the subjects German, mathematics, physics, chemistry and biology) affects secondary school children in Germany. This rich data set allows us to include around 14,000 9$^{th}$grade students in our analyses.

In this contribution, we rely on a grade equation approach [17, 63], assuming that discrepancies between observed grades and achievement as measured in standardised tests are evidence of biased grading. While one-time standardised tests are certainly not the most accurate measure of ability, we argue that they are nonetheless valuable in that they are more objective than grades since the evaluation of these tests is highly standardised and anonymous. Although—due to a lack of teacher-level data—we do not test mechanisms leading to the expected grading bias (e.g. stereotypes), we assume that teachers' perceptions of students' abilities can be biased one way or another depending on the characteristics of the students, the subject in question and their in-classroom behaviour.

We focus on three research questions: How severe/prevalent is the problem of grading bias by gender, socio-economic status (SES), ethnic background and weight status in Germany? Are there differences by subject and how large are these? Are there groups that are particularly negatively affected by grading bias because of overlapping disadvantaging factors? In other words: Does chubby Can get lower grades than skinny Sophie?

In the next section we give some context on the school systems in Germany before giving a brief overview of the current state of research as well as our theoretical considerations. This is followed by a description of the used data, our operationalisations as well as the analytical approach. We conclude by presenting and discussing the results.

## Background

### Grades in Germany

In Germany, the organisation of education is the responsibility of the federal states, resulting in 16 different education systems. Despite their differences, these systems share some organisational similarities. After kindergarten and primary school (in grade four or grade six depending on the federal state), students in Germany are selected into different secondary school tracks. In most federal states this system is three-tiered and consists of a track with lower requirements (*Hauptschule*) for years 5 to 9, a track with higher requirements (*Realschule*) for years 5 to 10 and an academic track (*Gymnasium*) for years 5 to 12 or 5 to 13 depending on the federal state. Typically students go to separate schools depending on their track. Alternatively, it is also possible for students to go to the same school up until year 10 (*Gesamtschule*), with some continuing on to the academic track. Successful completion of the academic school track is a requirement for admission to a university (of applied sciences). Students are graded on a scale from 1 for excellent to 6 for insufficient achievement at their respective levels, i.e. depending on their track.

## Existing evidence of grading bias

Numerous studies across various contexts and subjects (mostly mathematics and languages) have found that girls receive better grades than boys for the same standardised test performance (for Israel [43]; for Denmark [52]; for Spain [30, 51]; for the US [10, 25]; for Czechia [11]; for Germany [12]; for Italy [49, 50]; for France [9]; for Switzerland [27]; for Greece [46]; for Portugal [47]; for New Zealand [35]). However, there are also studies that do not find such a bias (for Sweden [64]; for India [26]). A study using the same data from Germany as we use here, but examining younger children, finds a grading bias in favour of girls in German and in favour of boys in mathematics [17]. Moreover, the results of some experimental studies suggest that math teachers rate boys more favourably [48]. In an experiment on gender bias regarding STEM subjects in Germany, Austria, and Switzerland, a clear pattern of bias against girls emerges. However, in this experiment, the teachers' biases disappears as they gain more experience teaching. Furthermore, no evidence was found for gender bias in the subject German [44]. Other experimental studies in Germany and Spain find no gender bias in teachers' evaluations of essays [31, 65].

Furthermore, quite a number of studies have documented grading bias or discrimination by ethnic origin, minority status or migration background (e.g. for Italy [13, 28]; for Brazil [34]; for UK [32]; for Spain [30, 31]; for the US [25, 38]; for Germany [12, 33, 37]; for New Zealand [35]). However, there are also studies suggesting that—given the same standardised test scores—(some) ethnic minority students are even given more favourable grades than ethnic majority students (for Denmark [52]; for the UK [32]) or graded similarly (for Germany [17, 65]; for Switzerland [27]).

Compared to the body of research on grading bias due to gender and ethnicity or migration background, there are significantly fewer studies on the influence of social background and weight status. Several studies indicate the existence of grading bias against socially disadvantaged children, more frequently observed in languages than in mathematics (for Italy [28]; for Switzerland [27]; for Germany [17]; for Spain [30, 31]; for India [26]; for the US [25]). In a German survey of teachers, children, and parents it was shown that teachers subconsciously gave higher grades to children from higher social classes than was justified by their actual competence levels. This overestimation was caused by teachers perceiving these pupils as "more talented, more willing to achieve and better equipped with parental resources than children from lower social classes" [29, p.1]. In another experiment where teachers grade essays, no evidence was found for discrimination in grading across gender, ethnicity and social class. However, teachers were found to have lower expectations of lower class and minority pupils' future performance in the case of higher-quality essays [65]. There also appears to be a bias against pupils who are overweight or obese (for the US [40–42]; for Sweden [39]; for Germany [17]).

## Potential mechanisms explaining grading bias

There are two dominant related explanatory approaches for grading bias: student behaviour in the classroom [11, 54, 55] and the stereotypes teachers hold of different groups [29, 37, 53].

Following the first line of reasoning, teachers' evaluations or grading is influenced by student behaviour in the classroom. For example, boys are often more disruptive, less self-disciplined, and less self-regulated than girls. This can result in lower grades [49, 66, 67] either through teacher perceptions and possible prejudice or by incorporating student behaviours in their grading schemes. Studies indicate that teachers give higher grades to students who are more well-adjusted and lower grades to students with more challenging behaviour [54]. Contrary to expectations, a study using data from Germany finds that overweight boys were more affected by bias than overweight girls. The authors suspect differences in classroom behaviour,

which they could not account for, to be the reason for this result [17]. A study on gender-bias in grading using data from Czechia offers support for this hypothesis and suggests non-cognitive skills as an explanation for the grading bias in favour of girls across the performance distribution (math and native language) [11]. The authors argue that non-cognitive skills as well as in-class and homework behaviour confound teacher assessments but not test scores. This interpretation is further strengthened by evidence from a non-experimental study from the US in which the disadvantage of boys (after controlling for test scores) in reading, math and science mostly disappears, and under some specifications even leads to a bonus, when non-cognitive skills are taken into account [10]. This stresses the importance of conforming behaviour at school. Evidence from a factorial survey experiment on teacher recommendations for secondary school in Switzerland suggests that challenging behaviour in class may be especially harmful to girls precisely because it is gender stereotype non-conforming [68].

The second main mechanism behind grading bias is that teachers' grading is influenced by stereotypes and thus expectations about children with different ascriptive characteristics. Implicit stereotypes have been suggested as the reason behind gender bias in the grading behaviour of math teachers to the detriment of girls in Israel [48] as well as in England, the US and Germany [8]. Similarly, in regard to language skills, boys have been found to be underestimated [8]. Research suggesting that gender bias against girls in STEM subjects disappears for more experienced teachers [44] speaks to the plausibility of this assumption.

In regard to ethnicity or migration background, it has been shown that teachers have stronger implicit stereotypes towards (Turkish) minority students (for Germany, [36, 69]) and have lower expectations of and less positive opinions about minority students (for the Netherlands, [53]). Teachers also seem to have higher expectations of European American and Asian American students compared to African American and Latinx students in the US [70], and teachers in Germany rate ethnic minority students who were described as stereotype-confirming lower in language proficiency but not in math in an experiment [71].

It has also been shown that teachers show a positive bias when evaluating the ability of students from more socio-economically privileged homes and are negatively biased in the case of less privileged pupils (experiment in the US: [24]). Furthermore, teachers' implicit stereotypes could be influenced by pressure from high SES parents, potentially affecting their beliefs about the academic potential of the students [13]. In a German study, the overestimation of students from high SES families was explained by teachers' more favourable perceptions of these pupils' attributes and resources [29]. Moreover, research on track recommendations also shows a bias against children from lower social classes [72, 73].

When it comes to weight bias, one of the first studies suggesting that perceptions of ability might also be affected by physical appearance showed that students give higher ratings to (fictitious) other students' essays if they are physically attractive and lower ratings if they are perceived as less attractive [74]. More recent studies have suggested that teachers' prejudices may be responsible for the grading bias against overweight children [40, 75] with teachers believing that overweight children have to work harder for the same results, end up having lower grades, and require more support [42].

## Grading bias from an intersectional perspective

To account for the fact that people can suffer from cumulative disadvantage by simultaneously facing obstacles on multiple grounds, or being unequally affected by different axes of inequality [76] depending on other aspects of their identities or group membership, we follow an intersectional approach [56, 77]. The term intersectionality was introduced by lawyer Kimberle Crenshaw in a discrimination lawsuit arguing that Black women had not been fired

because they were women or because they were Black but specifically for being Black women [56, 77]. Similarly, research on educational inequalities is increasingly taking intersectionality into account to do justice to the fact that depending on their unique combination of ascriptive characteristics, students can face cumulative (dis)advantage on multiple grounds [57–61]. However, to date, empirical research on grading bias taking intersectionality into consideration has been scarce, which is why we aim to contribute to this line of research [17, 31, 52]. More broadly however, it has e.g. been shown that Black girls in US schools are confronted with specific persistent negative stereotypes by teachers [61], as are male Muslim boys in Germany [78]—this could translate into biased grading. Teachers also seem to evaluate obese girls more negatively than boys in the US [75], while the opposite is true for Germany, where teachers especially penalise overweight boys in math [17]. These results suggest that stereotypes towards people with one ascriptive characteristic (e.g. gender) can be amplified by the presence of another ascriptive characteristic (e.g. overweight).

Furthermore, so far, research on intersectionality has mostly focused on cumulative (dis) advantage, which can be either additive in nature, or alternatively also entail an additional penalty or reward. However, it is of course also possible that the advantageous or disadvantageous effects certain aspects of one's identity can have in regard to an outcome cancel each other out and thus lead to no deviations from the average. This is e.g. the case when having a high social status cancels out the negative effect of being a migrant. In a large-scale factorial survey experiment on stereotypes focusing on the five social categories: gender, sexual orientation, age, ethnic and social class background, the intersections between these categories were found to be responsible for a large part of the variation in warmth and competence ratings. While most of the variation was caused by main effects for competence stereotypes, the opposite was true for warmth stereotypes [79]. Similarly, research on gender and ethnic (5 groups: Asian Americans, Blacks, Latinos, Middle Eastern Americans and white Americans) stereotypes using a free-response procedure has shown that the "gender-by-ethnic stereotypes" were not the result of adding up the respective parts but often contained unique elements specific to the combination in question [80].

In the context of grading bias, next to cumulative disadvantage, it is also possible that teachers would try to compensate particularly or multiply disadvantaged children by using a more lenient grading scheme towards them [31, 52]. In any case, depending on the strength of the stereotypes of the single attributes in question and the unique combinations of social categories, there are many possible scenarios as to what the overall effect on grading bias could be (from additional penalties to neutralisation of penalties to reversal, in extreme cases).

## Methods

### Data

In this contribution, we use data from the fourth starting cohort (SC4, 13.0.0, first survey in year 9 in 2010) of the *National Educational Panel Study* (NEPS). Our data set thus consists of a nationally representative sample of more than 14,000 ninth graders attained by a cluster sampling strategy (strata, schools and classes). For a more detailed description of the sampling strategy and the study design see [81]. The NEPS is carried out by the Leibniz Institute for Educational Trajectories (LIfBi, Germany) in cooperation with a nationwide network [82, 83]. Next to the questionnaires (paper-pencil-interviews) completed by the pupils in their classrooms, their parents were also surveyed (computer-assisted-interviews). In our contribution, we use data from the first two survey waves (at the beginning and end of year nine).

The NEPS study is conducted under the supervision of the German Federal Commissioner for Data Protection and Freedom of Information (BfDI) and in coordination with the German

Standing Conference of the Ministers of Education and Cultural Affairs (KMK) and—in the case of surveys at schools—the Educational Ministries of the respective Federal States. All data collection procedures, instruments and documents were checked by the data protection unit of the Leibniz Institute for Educational Trajectories (LIfBi). The necessary steps are taken to protect participants' confidentiality according to national and international regulations of data security. Participation in the NEPS study is voluntary and based on the informed consent of participants. This consent to participate in the NEPS study can be revoked at any time. Participant consent was individually obtained for this study, with legal representatives providing consent for minors. The Leibniz Institute for Educational Trajectories in Bamberg securely archives all consent documentation. To safeguard respondents, data usage is strictly limited to scientific research and is effectively anonymised. Prior to data sharing, the institutional data protection officer ensures scientific use and adequate anonymisation or protection.

For our analyses, we used fully anonymised archived samples of the NEPS data. We accessed the data for research purposes from September 2021 to March 2024. The last time we accessed the data was on 15 March 2024. While accessing the data, we did not have access to any information that could identify individuals, we only had access to fully anonymised data.

## Operationalisation

The most important dependent variables are the students' grades in all subjects. The data set supplies us with grades as well as achievement test results in German, mathematics, as well as the natural science subjects: biology, chemistry and physics. In Germany, the grading system uses numbers from 1 (excellent) to 6 (insufficient). For better readability, in our analyses we recode the grades so that the highest values signify the highest achievement and then z-standardise them. According to several studies in Germany, students self-report their grades fairly accurately [84, 85]. For the domain-specific competence in reading, mathematics, and the natural sciences, NEPS provides WLE-estimators. The tests were administered in the classroom and aim to measure competence over the life course (for more information on the competence tests, see [86]). Since the pupils perform the competence tests in different order, the WLE-estimators correct for the position of the respective test in the test booklet.

In addition, the NEPS also measures general cognitive basic skills as cross-domain competence using the DGCF (Domain General Cognitive Functions) test. In the data, the test results are available as sum scores for reasoning and perceptual speed.

To capture differences between students with and without a migration background, we use two different operationalisations. First, we distinguish between ethnic majority and ethnic minority students, without differentiating between the various ethnic origins of the minority students. We classify students as ethnic minority students if they are first to 3.5 minority generation (minority status). Second, following previous literature and due to the available case numbers, we differentiate between the following places of origin: (1) Turkey, (2) the former Soviet Union (FSU), (3) Northwestern and Southern Europe, (4) Central and Eastern Europe as well as (5) other countries (minority group). Assignment to minority status and group is based on the countries of birth of the parents and grandparents using the origin group variable provided by the NEPS.

To classify body weight, we calculate the BMI using the students' height and body weight. This value is then assigned a percentile value from the Center for Disease Control and Prevention (CDC) classification table according to the age (in months) of the students [87]; for a similar procedure, see [17]. Students were classified as overweight if they had a percentile score of 85 to less than 95 and as obese if they had a percentile score greater than or equal to 95. Due to the small number of students meeting these criteria, we decided to create a binary indicator

(overweight or obese) that takes a value of 0 if students have a percentile score less than 85 and a value of 1 if students have a percentile score greater than or equal to 85.

For the operationalisation of social origin, we used the highest ISEI [88] of the parents. The ISEI can take on values between 11 and 90. This variable is based on information the pupils gave about their parents' occupations. For cases in which the pupils did not provide information about their parents' jobs, we rely on the information from the parent interviews where possible. We primarily rely on the information the students provide because of the higher percentage of complete questionnaires compared to the parent interviews. For the descriptive analyses, we subdivide the student population into deciles of socio-economic status. For the regression analyses, we use the z-standardised highest ISEI of the parents. To control for psychological characteristics and in-classroom behaviour, we use the following variables: the "big five", the *Strengths and Difficulties Questionnaire* scale (SDQ) [89], health satisfaction, the *Sick, Control, One, Fat and Food* (SCOFF) scale [90] and a binary indicator for class retention.

The big five scales were generated using the answers to two items respectively:

1. openness: 1) "I have a vivid imagination, I am an imaginative person." 2) "I have little interest in artistic things." (recoded)
2. conscientiousness: 1) "I am easy-going and tend to be a bit lazy." (recoded) 2) "I am thorough when completing my tasks."
3. extraversion: 1) "I am quite cautious, reserved." (recoded) 2) "I am out-going and sociable."
4. agreeableness: 1) "I trust other people easily, I believe in the good in people." 2) "I tend to be critical of other people."
5. neuroticism: 1) "I am relaxed and don't get stressed easily." 2) "I am considerate towards others, sensitive."

The scale values were z-standardised for analysis. Furthermore, we use two SDQ scales: one for measuring prosocial behaviour and the other for measuring problems with peers. Both scales can take values between 0 and 10. An example of an item from the *prosocial behaviour* scale is: "I try to be nice to other people. I care about their feelings." An example of an item from the *problems with peers* scale is: "I am usually on my own. I generally play alone or keep to myself." Once again, the scale values were z-standardised for analysis. Health satisfaction was measured using the following item: "How satisfied are you with your health?" using a scale ranging from 0) "completely dissatisfied" to 10)"completely satisfied". The scale values were also z-standardised for analysis. The SCOFF scale is a scale for measuring problematic eating behaviour [90]. One of the items is: "Do you make yourself sick because you feel uncomfortably full?". This scale forms a sum score of the answers, taking values between 0 and 5, with higher values indicating problematic eating behaviour. The dummy variable for class retention is an indicator of whether a student repeated a grade at least once by the time of the survey.

Furthermore, we control for the different school types in the German education system: the secondary school track with lower (*Hauptschule*) requirements, the track with higher (*Realschule*) requirements, the academically oriented secondary school (*Gymnasium*) as well as the comprehensive schools (*Gesamtschule*).

## Sample selection

Our analytical sample comprises 14,090 students who participated in the second wave of the survey NEPS SC4 at the end of grade 9 and are not attending a special school [81]. S1 Table in the Supporting Information (SI) documents the number of cases, panel attrition and missing

values for key variables. Variables with the highest number of missing values are: ISEI (12.3%), BMI (15.5%) and school grades in physics (13.2%), chemistry (13.7%) as well as biology (18.7%). The high number of missing values in the grades for biology, chemistry and physics is due to the fact that not all students attend these subjects. Thus, all students who indicated that they did not receive a grade in a subject were excluded from the respective analysis. We end up with the following case numbers per subject: $N_{German}$ = 14,005, $N_{mathematics}$ = 13,964, $N_{physics}$ = 12,956, $N_{chemistry}$ = 12,898, $N_{biology}$ = 12,207.

## Multiple imputation

As shown in S1 Table, some model variables have a large proportion of missing values. We decided to deal with this problem by applying multiple imputation by chained equations to generate 50 multiple imputed data sets without missing values (predictive mean matching using burnin = 50). A separate imputation model was calculated for each sample/subject. In addition to all model variables, the imputation models also include the following auxiliary variables: school grades in the other subjects, domain-specific test scores for the other subjects, school grades in mathematics and German from wave 1 (beginning of $9^{th}$ grade), self-esteem scale, job aspirations for age 30 (ISEI), parental education (CASMIN), subjective interest in mathematics and German (wave 2), life satisfaction (wave 1), school satisfaction (wave 1), the sampling stratum, as well as the student and school weights.

## Estimation strategy

Following other studies on this topic, we use the so-called grade equation approach [17, 63] to estimate grading bias. This means that we investigate to what extent ascriptive characteristics (BMI, gender, ethnic background, SES) have an influence on school grades while controlling for the results of standardised competence tests (here we use domain-specific as well as general cognitive tests) and potential mediator variables (psychological characteristics and classroom behaviour). This approach assumes that standardised competence tests are a more objective measure of domain-specific competence than school grades and that they thus provide an objective reference value for skills [12, 17]. A major advantage of using standardised achievement tests is that the results are obtained independently of the teachers who give the students their school grades. Additionally, the tests are evaluated by researchers who do not know the students. Thus, it can be assumed that there is no danger of bias by ascriptive characteristics in the standardised competence tests.

In our regression analyses, we proceed step by step. For each of the five different grades we calculate the following models: In a first analysis, we estimate how strong the grading bias is (model 1; see Eq (1)). In each of these models, we include the ascriptive characteristic of interest (e.g. gender or BMI status) as well as the respective domain-specific competence score (e.g. for mathematics, the WLE score in mathematics), the general cognitive ability test scores and the school track attended. However, it should be noted that for the subjects chemistry, biology and physics, we rely on a general competence test for the natural sciences. We thus obtain an estimator for the total effects of grading bias in each subject for the ascriptive characteristics of interest. Given the structure of our data, we estimate three-level multilevel models with the levels: students (i) nested within classes (j) nested within schools (j). To reflect possible contextual variations in grading, we include random intercepts at the school and class level in the models. Since we are interested in grading bias for 5 factors (gender, body-weight, social origin as well as minority status and group) in 5 subjects (German, mathematics, biology, chemistry and

physics), we estimate model 1, depicting the total effects of grading bias, 25 times.

$$Y_{ijk} = \gamma_{000} + \gamma_A A_{ijk} + \gamma_X \mathbf{X_{ijk}} + u_{0jk} + v_{00k} + \sigma_{ijk} \quad (1)$$

where

$Y_{ijk}$ is the z-standardised school grade;

$\gamma$ are the regression coefficients;

$A_{ijk}$ is one of the five ascriptive characteristics;

$\mathbf{X}_{ijk}$ is a vector of the individual-level model variables, including domain-specific test scores, general cognitive test scores and attended school track;

$u_{0jk}$ is the random intercept at the class level;

$v_{00k}$ is the random intercept at the school level;

$\sigma_{ijk}$ is the error term.

In a next step, we calculate models that also include the other ascriptive characteristics (taking into account either minority status or minority group) as well as all other control variables (model 2; see Eq (2)). Our goal is to assess to what extent psychological characteristics and behaviour contribute to the explanation of the observed grading bias. We estimate this model 10 times (5 times for each subject including minority status and 5 times for each subject including minority group).

$$Y_{ijk} = \gamma_{000} + \gamma_{\mathbf{A}} \mathbf{A}_{ijk} + \gamma_X \mathbf{X_{ijk}} + \gamma_Z \mathbf{Z_{ijk}} + u_{0jk} + v_{00k} + \sigma_{ijk} \quad (2)$$

where

$Y_{ijk}$ is the z-standardised school grade;

$\gamma$ are the regression coefficients;

$\mathbf{A}_{ijk}$ is a vector of the ascriptive characteristics;

$\mathbf{X}_{ijk}$ is a vector of the individual-level model variables, including domain-specific test scores, general cognitive test scores; and attended school track;

$\mathbf{Z}_{ijk}$ is a vector of the individual-level model control and mediator variables, including psychological traits and in-classroom behaviour;

$u_{0jk}$ is the random intercept at the class level;

$v_{00k}$ is the random intercept at the school level;

$\sigma_{ijk}$ is the error term.

In a third step, we test for intersectional effects of social origin, gender, body weight, and minority status or group on grading bias using several approaches. First, we estimate a model based on model 1 that includes all main effects of the above ascriptive characteristics, but no interactions between them. Second, we estimate the same model as in the first step, but including all two-way interactions between the four ascriptive characteristics. Third, we estimate a model that includes all 4-way interactions between the four ascriptive characteristics. Based on the results of these models, we calculate predictive margins of grades for different groups of students with different combinations of ascriptive characteristics and then compute the differences. This allows us to account for potential intersectional disadvantages by e.g. comparing low SES (-1 SD) overweight/obese boys of Turkish descent with high SES (+1 SD) non-overweight/obese majority girls.

## Results

### Grading bias

Fig 1 shows the average bivariate grade differences for the ascriptive characteristics: gender, BMI status, SES, minority status and minority group. Furthermore, it shows the results of two

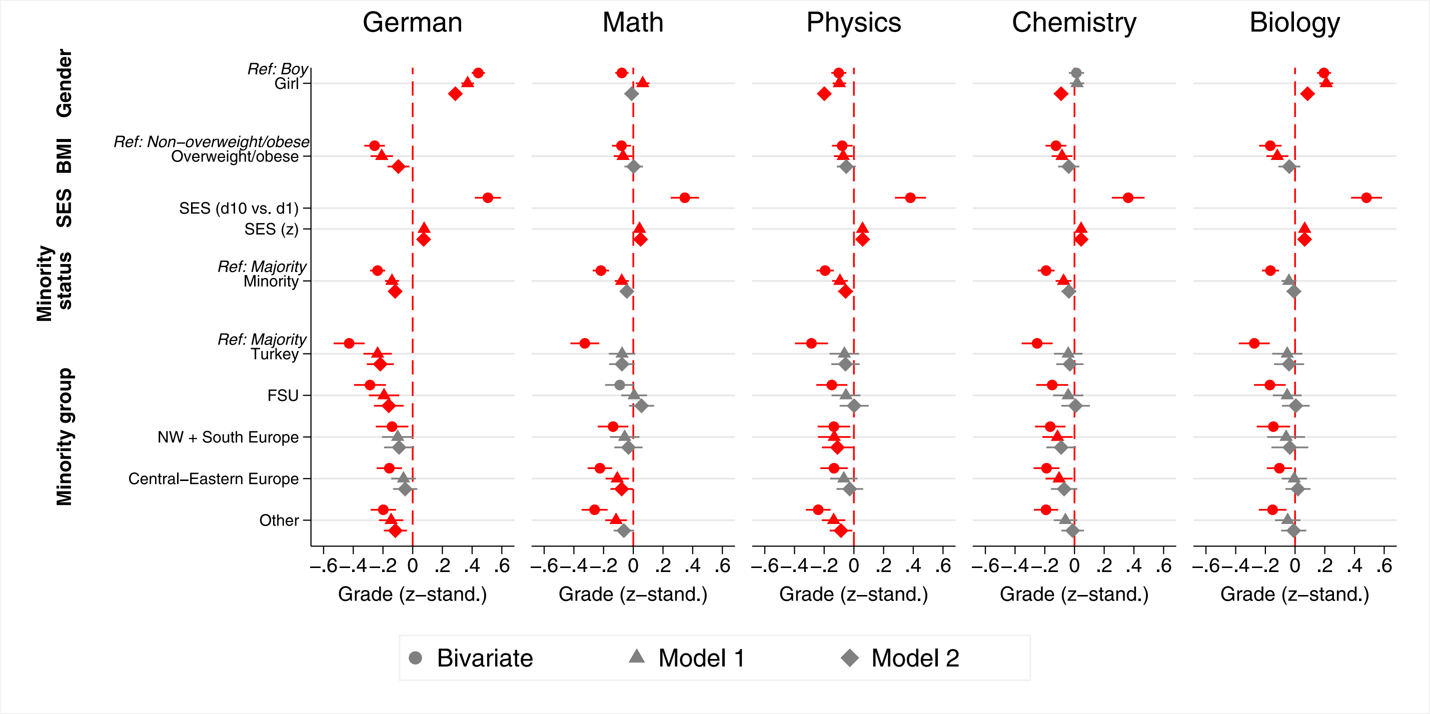


**Fig 1. Mean school grade differences and grading bias by students' ascriptive characteristics across subjects.** Note: Red coloured icons indicate statistical significance. Regression coefficients based on three-level linear regression models. Model 1 adjusts for domain-specific competence, general academic competence and school track. Model 2 adjusts for all model variables (see S2–S6 Tables). Source: NEPS SC4 (based on m = 50 multiple imputed datasets); weighted data, our own calculations.

https://doi.org/10.1371/journal.pone.0305703.g001

models: the total effects of the ascriptive characteristics (grading bias) on school grades (model 1) as well as to what extent this grading bias is influenced by psychological traits or in-classroom behaviour (model 2). The corresponding regression tables are shown in S2–S6 Tables. For a figure depicting the mean test score differences, see S1 Fig.

Regarding the bivariate differences, we see that, in some cases, there are considerable differences in the absolute school grades between these groups. For example, girls have significantly better grades in German (0.44 SD) and biology (0.19 SD) than boys while boys have slightly better grades in mathematics and physics. There is no significant gender difference in chemistry grades. Furthermore, overweight/obese students have lower grades than non-overweight/obese students in all subjects. This effect is least pronounced in mathematics and most pronounced in German and biology. We also see a considerable difference in grades, to the advantage of the more privileged students, between the first and the tenth SES deciles in all school subjects. Moreover, minority students have lower absolute grades than majority students. While these effects are relatively similar for all subjects, they are once again most pronounced in German. Almost all minority groups show lower grades than majority students in all subjects (except the FSU in mathematics). Among minority students, those from Turkey have the lowest grades. We therefore see the largest difference to the majority group for students from Turkey.

We see from the results of model 1 (see Fig 1) that students with different ascriptive characteristics receive different grades through almost all school subjects, even when they demonstrate the same domain-specific as well as general cognitive competence. In more detail, we see grading bias by gender in all subjects except for chemistry and girls seem to have an advantage

in German (0.37 SD), mathematics (0.06 SD) and biology (0.21 SD), while boys have an advantage in physics (-0.10 SD). Looking at the results for students with different body weight, we see that overweight/obese students receive lower grades through all subjects (from -0.21 in German to -0.07 SD in mathematics and physics). Similarly, students with higher parental SES receive higher grades in all subjects (from 0.08 in German to 0.04 SD in mathematics and chemistry). Furthermore, we see that minority students also have a disadvantage through all subjects (from -0.14 in German to -0.07 SD in chemistry) except biology. If we look at the minority groups separately however, we only find significant differences to the majority group in some cases and mostly in German, where we see a disadvantage for pupils from Turkey (-0.24 SD), the FSU (-0.19 SD) and "other" countries (-0.15 SD). In mathematics we see a disadvantage for pupils from Central-Eastern Europe (-0.11 SD) as well as from "other" countries (-0.12 SD), while in physics a disadvantage is found for pupils from North-Western and Southern Europe (-0.13 SD), as well as "other" countries (-0.14 SD). For chemistry, we find a disadvantage for students from North-Western and Southern European (-0.11 SD) as well as Central-Eastern European countries (-0.10 SD). No differences are found for biology.

A comparison of coefficients for girls and boys in model 2 (see Fig 1) shows that, in general, when controlling for personality traits and grade retention, boys seem to gain in grade points compared to girls. However, especially in German (0.29 SD) and biology (0.08 SD), we still see a large discrepancy to the advantage of girls. In mathematics there are no more significant differences in grades while in physics the advantage of boys is larger than in model 1 (-0.2 compared to -0.1 SD) and they now also have an advantage in chemistry (-0.09 SD). Regarding body weight, the inclusion of the personality traits and the indicator for grade retention reduces the disadvantages for overweight/obese students. Significant disadvantages remain only in German (-0.1 SD). However, the inclusion of the variables operationalising personality and behaviour does not affect grading bias by social origin. The advantages for socio-economically privileged students remain around the same magnitude in all subjects. Regarding effects of ethnic origin, we see that adding the additional variables leads to a reduction of the disadvantage for minority pupils. While, this reduction is not very pronounced for German and physics, the disadvantages for minority students in mathematics and chemistry are no longer apparent. However, in German, the disadvantages in school grades remain for all minority groups except for the European minorities—with the most pronounced disadvantage for students from Turkey (-0.22 SD). In the other subjects, disadvantages are still evident for pupils from Central-Eastern European countries (-0.08 SD) in mathematics, and students from North-Western and Southern European (-0.11 SD) as well as "other" countries (-0.12 SD) in physics. There are no (longer) any differences to be found by ethnic group in chemistry and biology. The results suggest that although psychological and behavioural factors do seem to have an impact on grading, the effect is not very large.

## Intersectional disadvantage?

To further illustrate the intersectional effects of the ascriptive characteristics of interest, we calculate predictive margins based on the above models including no interaction effects, 2-way interaction effects, and 4-way interaction effects. Fig 2 shows how much lower grades are for female and male minority students or those with a Turkish background (since this is the minority group where we see the biggest disadvantage in Fig 1) for different combinations of body weight and social origin. The corresponding regression tables are shown in S7–S11 Tables. For a figure depicting the predictive margins of majority students compared to minority students, see S2 Fig.

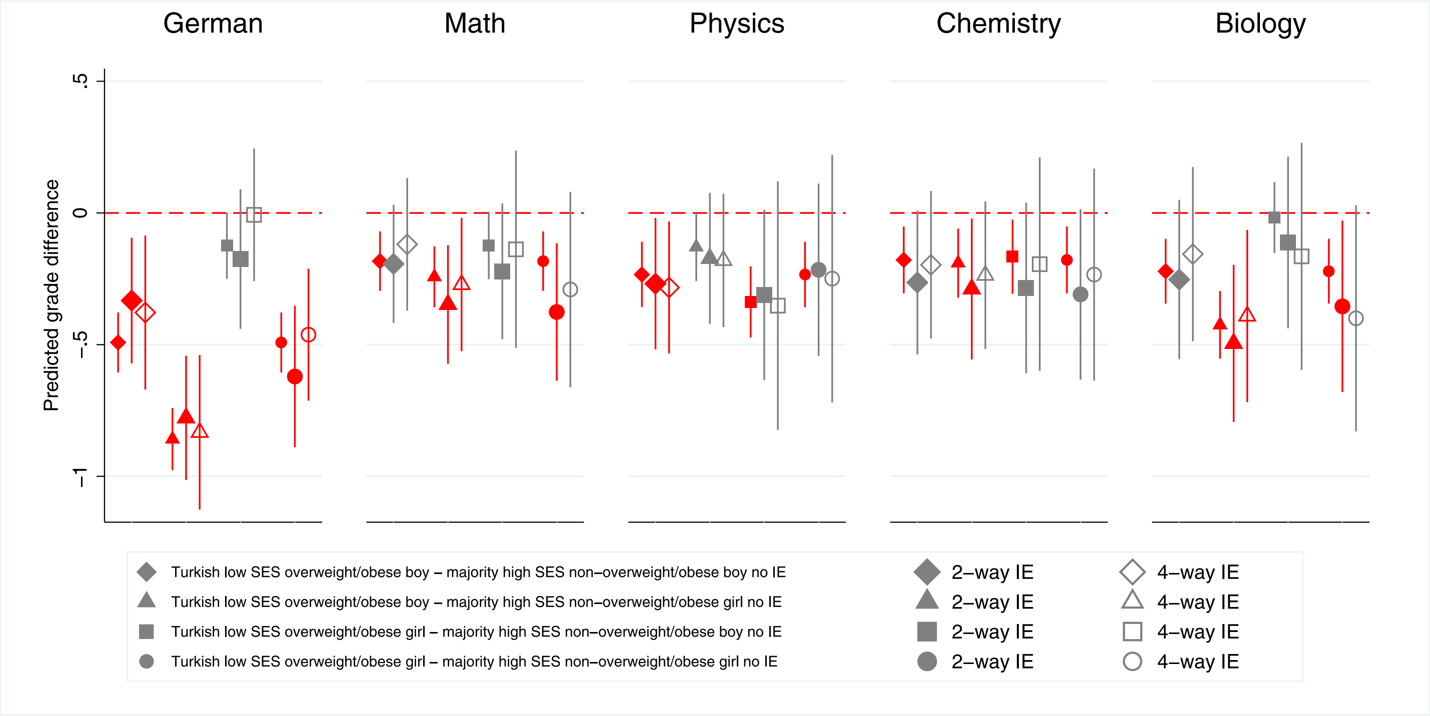


**Fig 2. Predicted school grade differences for students with specific ascriptive characteristics across subjects and different models.** Note: Red coloured icons indicate statistical significance. Predictive margins at the means of all other model variables. Predictions derived from multilevel regression models (see S7–S11 Tables). No IE = models without interactions, 2-way IE = models with all 2-way interactions, 4-way IE = models with all 4-way interactions. Source: NEPS SC4 (based on m = 50 multiple imputed datasets); weighted data, our own calculations.

https://doi.org/10.1371/journal.pone.0305703.g002

The results suggest that the different models (no interaction effects, 2-way interaction effects, and 4-way interaction effects) lead to fairly similar predicted grade differences. Although the confidence intervals in the interaction models are significantly wider, making the predicted grade differences less likely to be statistically significant, the point estimates are often quite similar. These results suggest that the negative effects of ascriptive characteristics accumulate. Thus, we find evidence of an additive intersectional disadvantage, but no evidence of a significant additional penalty or strong compensation beyond the additive effects, since the interaction models yield very similar results to the models without interactions. This interpretation, based on the predicted grade differences, is further supported by the fact that very few of the 2-way or 4-way interactions are statistically significant (see, S7–S11 Tables). In German and mathematics, some combinations of girl and high-SES show a small positive effect. However, no consistent patterns can be derived from the few significant interaction effects.

To sum up, even though we do not find evidence of additional penalties or compensation effects beyond the sum of individual effects of gender, body weight as well as ethnic and social background, significant differences between groups are evident. Generally, the grade penalties are most substantial in the subjects German and biology. Moreover, the grade penalty is particularly pronounced for Turkish overweight/obese boys from socio-economically disadvantaged families compared to majority non-overweight/obese girls from advantaged families in German. With equal domain-specific and general proficiency scores, these students receive a German grade that is approximately 0.8 SD lower. We observe similar, though less dramatic, grade penalties across all subjects for Turkish overweight/obese boys and girls from socio-economically disadvantaged families. Looking at the predictive margins for all ethnic minority students

compared to majority students (see, S2 Fig), similar patterns emerge. However, since the number of cases is much bigger (comparing all ethnic minority students to majority students), the confidence intervals are smaller and we find statistically significant differences for more combinations of ascriptive characteristics.

### Robustness checks

To test the robustness of our results, we take a few measures. First, we transform our dependent variable (the school grades) differently. Instead of using z-standardised grades, we use the grade ranks of students within a class as the dependent variable and run linear regression models with clustered standard errors on the class level. We thus create grade ranks for each school class and standardise them to values between 0 and 1. To avoid possible biases that might occur in small classes where it might be difficult to create ranks, we restrict our analysis sample by only including students in classes with at least 10 pupils. While due to the smaller sample and the transformed value range (ranks take values between 0 and 1) we observe statistically significant results less often, the results for model 1 and model 2 are very similar to the results presented above using the z-standardised scores (see S3 Fig). Again, the effects are most pronounced in German. Furthermore, the gender effects seem to be very stable in all subjects.

Second, we let go of the assumption that the effects of the ascriptive characteristics of interest are constant across classes [52]. We therefore estimate random-intercept, random-slope models for model 1 (instead of random-intercept models), thus allowing the effects of the ascriptive characteristics to vary across classes. We chose to restrict our analysis sample based on relevant characteristics of the class composition: To be included, the class had to consist of at least 10 students, and there also needed to be at least one child with and one child without the specific ascriptive trait of interest per class, e.g. at least one boy and one girl. Since there are many classes without at least one child from each minority group, we do not estimate random slopes for the separate minority groups but use minority status instead. The models are computed using only complete cases (CCA), since it is not possible to display the distribution of random slopes in a multiple imputation (MI) framework. The switch from MI to CCA should not be a problem, since the effect estimates we are interested in are very similar in both approaches (see S4 Fig).

The patterns of the distribution of slopes across school classes in the random-slope models (see S5–S9 Figs) are worth noting. While there is not much variance across classes regarding the positive effects of a higher socio-economic background on grades through all subjects, the effects of the other ascriptive characteristics vary considerably in magnitude depending on the class. Especially in regard to gender and body weight, we see a large variance in effect size, while the differences are somewhat less pronounced for minority status. This means that the disadvantage some students face due to their gender, weight, socio-economic as well as ethnic background are stronger depending on where they go to school. However, without additional analyses, we cannot say what the cause of these differences is. All in all, the results of our robustness analyses are reassuring.

## Discussion & conclusion

In this paper, our primary goal was to develop an understanding of grading bias by gender, body weight as well as social origin and ethnic background in German secondary schools from an intersectional point of view. More to the point, we set out to see whether Skinny Sophie receives higher grades than chubby Can, even if they have the same skills and are equally intelligent. Our analyses on grading bias in five subjects has shown this to be overwhelmingly the case.

For our analyses, we rely on observational data that simultaneously supplies us with grades and results of various competence tests for thousands of secondary school students in Germany and for five subjects including German and mathematics. Furthermore, while existing research tends to focus on grading bias for minority students or on gender effects [33, 48, 71], we additionally test for disadvantages due to body weight and socio-economic status.

After looking at the bivariate relationships between the ascriptive characteristics and grades, we test whether the group differences can be explained fully by actual differences in skills. For this purpose, we estimate a model including not only the ascriptive characteristics of interest, but also the results of the respective domain-specific competence tests, the scores of two general academic competence tests (reasoning and perceptual speed) and the school track (model 1 in Fig 1). As Fig 1 shows, the grade differentials mostly remain even after controlling for the ability of the students using these three separate measures. Furthermore, even after adding further variables that might have an influence on grades, such as personality traits as well as grade retention as an indicator of classroom behaviour (model 2 in Fig 1), most differentials remain stable [50]. We interpret these findings as a strong indicator of an existing grading bias affecting different groups of students depending on their gender, body weight, socio-economic as well as ethnic background in German secondary schools across all five subjects included in the analyses.

Since pupils can be affected by grading bias on multiple grounds simultaneously, we compare group differences for students with specific combinations of characteristics. For example, we expected the grades of an overweight Turkish boy from a less privileged socio-economic status (SES) to be cumulatively biased in German compared to a non-overweight German girl from a privileged social background. To test for these potential intersectional effects, we thus calculated the predictive margins for different combinations of ascriptive characteristics on the basis of model 1 including either no interaction effects, 2-way interaction effects, or 4-way interaction effects between the 4 axes of inequality (Fig 2). While there are only few significant interaction effects, suggesting a (mostly) additive interplay between the factors, comparing the predicted mean grades for Turkish, low-SES, overweight/obese boys and girls with majority, high-SES, non-overweight/obese boys and girls exemplifies the existing intersectional (dis) advantages. We find significant differences in the predicted grades in many cases—because of the larger case numbers, this is especially true when comparing all minority groups combined to majority pupils. We find the largest effect sizes for the subject German. A potential explanation for this finding is that teachers presumably have more freedom when evaluating language skills (e.g. an essay or presentation) than skills in mathematics or the natural sciences where there is a clearer distinction between a right and a wrong answer. In support of this interpretation, several studies showed that grading bias by ethnic background is larger when the evaluation criteria were more vague [37, 38]. All in all, our findings strongly suggest that grading bias is widespread and that students are affected by intersectional inequalities.

We also performed two robustness checks and the results back up our main findings. Using a different measure for grades and using different model specifications to predict the grade differences for specific subgroups yielded no substantial differences to the approach used in the main analyses. To test whether the disadvantages we find (model 1) are due to individual class differences (specific teachers) or whether they can be observed more globally, we computed random slope models (S5–S9 Figs) as an additional robustness test. These models show that the observed grading bias cannot be traced back to a few classes/teachers and that on the contrary, it seems a common phenomenon. However, there does seem to be quite some variance in the degree of the grading bias that also depends on the ascriptive characteristics of interest [52]. While there seems to be only a small degree of variance in the effect of socio-economic

background, the ways in which gender and body weight influence grades seems to be much more context sensitive.

A limitation of our study is that, even though we use a large data set, some groups of interest, and especially the cross-sections of the groups (e.g. minority, low-SES, obese/overweight girl) are not very highly represented. The wide confidence intervals, especially for interactions and predictive margins, may be due to the low case numbers for these groups. This could be a reason why we are largely unable to identify robust patterns of significant interaction effects between the characteristics. Furthermore, also due to the small subsample-sizes, we did not differentiate between different migrant generations although this could also be a relevant factor [91]. Future research on the topic could thus benefit from using administrative data as has been done for Denmark [52]. A further limitation of the study is that, by assuming that the difference between grades and test scores is due to teachers' biased grading, we exclude possible alternative explanations such as actual performance differences induced by stereotype threat [92–94]. Since we do not examine the causes for grading bias, but merely try to illustrate the prevalence and degree of the bias, as well as show who is most affected by it, further research on the subject is still needed for a better understanding of the phenomenon.

Knowing more about the cause(s) of grading bias would definitely be helpful for designing effective policy measures aiming to combat it and thus create a slightly more just school environment. Nonetheless, our contribution offers ample evidence for wide-spread grading bias in the German school system, affecting pupils with a lower socio-economic background, minorities and those who are overweight or obese. We also find grading-bias by gender, however depending on the subject, girls or boys are negatively affected. Moreover, there seems to be a cumulative disadvantage for students who simultaneously belong to more than one group facing disadvantage. While our findings suggest that psychological measures and thus in-classroom behaviour of students may affect the evaluations of teachers, these effects seem to be marginal. However, if grades are supposed to be a measure of a students' skills in the subject in question, then—controlling for ability—the way they behave in class should have no additional impact on the grade in their reports. Having a discussion on the role of grades and what they are meant to be a measure of could thus contribute to a system that distributes grades more accurately and thus fairly. Furthermore, it has been shown that when using a clearly defined rubric for grading students' work, the racial-bias in teachers' grading can be corrected somewhat [38]. Our contribution, which uncovers the highest degree of grading bias for German, also suggests that the more freedom teachers have in grading their pupils, the more likely it is that different forms of bias will affect their evaluations. Implementing more structured grading schemes could therefore contribute to fairer grades.

## Supporting information

**S1 Fig. Mean domain-specific test score group differences across subjects.**
(PDF)

**S2 Fig. Predicted school grade differences for students with specific ascriptive characteristics across subjects and different models.** Comparing majority to minority students.
(PDF)

**S3 Fig. Student rank grading bias by students' ascriptive characteristics.**
(PDF)

**S4 Fig. Effect comparison (model 1) of the random-intercept and random-intercept random-slope models.**
(PDF)

**S5 Fig. Distribution of the effects slopes of the ascriptive characteristics (model 1) on German grade.**
(PDF)

**S6 Fig. Distribution of the effects slopes of the ascriptive characteristics (model 1) on math grade.**
(PDF)

**S7 Fig. Distribution of the effects slopes of the ascriptive characteristics (model 1) on physics grade.**
(PDF)

**S8 Fig. Distribution of the effects slopes of the ascriptive characteristics (model 1) on chemistry grade.**
(PDF)

**S9 Fig. Distribution of the effects slopes of the ascriptive characteristics (model 1) on biology grade.**
(PDF)

**S1 Table. Information on potential sample sizes, sample sizes and missing value patterns.**
(PDF)

**S2 Table. Multilevel-linear regression results (regression coefficients and [95% confidence intervals]) predicting school Grades in German (models 1 + 2).**
(PDF)

**S3 Table. Multilevel-linear regression results (regression coefficients and [95% confidence intervals]) predicting school Grades in Math (models 1 + 2).**
(PDF)

**S4 Table. Multilevel-linear regression results (regression coefficients and [95% confidence intervals]) predicting school Grades in Physics (models 1 + 2).**
(PDF)

**S5 Table. Multilevel-linear regression results (regression coefficients and [95% confidence intervals]) predicting school Grades in Chemistry (models 1 + 2).**
(PDF)

**S6 Table. Multilevel-linear regression results (regression coefficients and [95% confidence intervals]) predicting school Grades in Biology (models 1 + 2).**
(PDF)

**S7 Table. Multilevel-linear regression results (regression coefficients and [95% confidence intervals]) predicting school Grades in German (Intersectional models).**
(PDF)

**S8 Table. Multilevel-linear regression results (regression coefficients and [95% confidence intervals]) predicting school Grades in Math (Intersectional models).**
(PDF)

**S9 Table. Multilevel-linear regression results (regression coefficients and [95% confidence intervals]) predicting school Grades in Physics (Intersectional models).**
(PDF)

**S10 Table. Multilevel-linear regression results (regression coefficients and [95% confidence intervals]) predicting school Grades in Chemistry (Intersectional models).**
(PDF)

**S11 Table. Multilevel-linear regression results (regression coefficients and [95% confidence intervals]) predicting school Grades in Biology (Intersectional models).**
(PDF)

**S1 File.**
(ZIP)

## Acknowledgments

We would like to thank Tamara Gutfleisch for helpful comments on earlier versions of this paper. We have presented this paper at several conferences (Venice Seminar on Analytical Sociology in 2021, Research Colloquium in Sociology at the University of Bern in 2021, 2022 ECSR Conference in Amsterdam, and the 2022 NEPS Online Conference) and thank the participants for their comments, which helped us to improve the paper. Furthermore, we would like to thank the editor and the two reviewers for their valuable suggestions that helped improve the manuscript.

## Author Contributions

**Conceptualization:** Richard Nennstiel, Sandra Gilgen.

**Data curation:** Richard Nennstiel.

**Formal analysis:** Richard Nennstiel.

**Investigation:** Richard Nennstiel, Sandra Gilgen.

**Methodology:** Richard Nennstiel, Sandra Gilgen.

**Project administration:** Richard Nennstiel, Sandra Gilgen.

**Supervision:** Richard Nennstiel, Sandra Gilgen.

**Visualization:** Richard Nennstiel.

**Writing – original draft:** Richard Nennstiel, Sandra Gilgen.

**Writing – review & editing:** Richard Nennstiel, Sandra Gilgen.

## References

1. Becker R, Lauterbach W, editors. Bildung als Privileg: Erklärungen und Befunde zu den Ursachen der Bildungsungleichheit. Springer VS.; 2013.
2. Busse R, Michaelis C, Nennstiel R. To what extent do secondary effects shape migrants' educational trajectories after lower-secondary education? Empirical Research in Vocational Education and Training. 2023; 15(1):3. https://doi.org/10.1186/s40461-023-00142-0
3. Nennstiel R. Do more demanding lower secondary school certificates for minority students pay off? A comparison of VET access between Germany and German-speaking Switzerland. SozW Soziale Welt. 2021; 72(3):313–342. https://doi.org/10.5771/0038-6073-2021-3-313
4. Sinclair S, Nilsson A, Cederskär E. Explaining gender-typed educational choice in adolescence: The role of social identity, self-concept, goals, grades, and interests. Journal of Vocational Behavior. 2019; 110:54–71. https://doi.org/10.1016/j.jvb.2018.11.007
5. Thompson ME. Grade Expectations: The Role of First-Year Grades in Predicting the Pursuit of STEM Majors for Firstand Continuing-Generation Students. The Journal of Higher Education. 2021; 92 (6):961–985. https://doi.org/10.1080/00221546.2021.1907169

6. Kalter F. Auf der Suche nach einer Erklärung für die spezifischen Arbeitsmarktnachteile von Jugendlichen türkischer Herkunft: Zugleich eine Replik auf den Beitrag von Holger Seibert und Heike Solga: Gleiche Chancen dank einer abgeschlossenen Ausbildung? (ZfS 5/2005). Zeitschrift für Soziologie. 2006; 35(2):144–160. https://doi.org/10.1515/zfsoz-2006-0204
7. Bergold S, Steinmayr R. Teacher Judgments Predict Developments in Adolescents' School Performance, Motivation, and Life Satisfaction. Journal of Educational Psychology. 2023; 115(4):642–664. https://doi.org/10.1037/edu0000786
8. Olczyk M, Gentrup S, Schneider T, Volodina A, Perinetti Casoni V, Washbrook E, et al. Teacher judgements and gender achievement gaps in primary education in England, Germany, and the US. Social Science Research. 2023; 116:102938. https://doi.org/10.1016/j.ssresearch.2023.102938 PMID: 37981394
9. Terrier C. Boys lag behind: How teachers' gender biases affect student achievement. Economics of Education Review. 2020; 77:101981. https://doi.org/10.1016/j.econedurev.2020.101981
10. Cornwell C, Mustard DB, Van Parys J. Noncognitive skills and the gender disparities in test scores and teacher assessments: Evidence from primary school. Journal of Human Resources. 2013; 48(1):236–264. https://doi.org/10.3368/jhr.48.1.236
11. Protivínský T, Münich D. Gender Bias in teachers' grading: What is in the grade. Studies in Educational Evaluation. 2018; 59:141–149. https://doi.org/10.1016/j.stueduc.2018.07.006
12. Kiss D. Are immigrants and girls graded worse? Results of a matching approach. Education Economics. 2013; 21(5):447–463. https://doi.org/10.1080/09645292.2011.585019
13. Triventi M. Are children of immigrants graded less generously by their teachers than natives, and why? Evidence from student population data in Italy. International Migration Review. 2020; 54(3):765–795. https://doi.org/10.1177/0197918319878104
14. Lindberg L, Persson M, Danielsson P, Hagman E, Marcus C. Obesity in childhood, socioeconomic status, and completion of 12 or more school years: A prospective cohort study. BMJ Open. 2021; 11(3): e040432. https://doi.org/10.1136/bmjopen-2020-040432 PMID: 33707266
15. Averett SL, Stifel DC. Race and gender differences in the cognitive effects of childhood overweight. Applied Economics Letters. 2010; 17(17):1673–1679. https://doi.org/10.1080/13504850903251256
16. Clark D, Slate JR, Viglietti GC. Children's weight and academic performance in elementary school: Cause for concern? Analyses of Social Issues and Public Policy. 2009; 9(1):185–204. https://doi.org/10.1111/j.1530-2415.2009.01186.x
17. Dian M, Triventi M. The weight of school grades: Evidence of biased teachers' evaluations against overweight students in Germany. PlOS ONE. 2021; 16(2):e0245972. https://doi.org/10.1371/journal.pone.0245972 PMID: 33556097
18. Marx AE, Stanat P. Reading comprehension of immigrant students in Germany: Research evidence on determinants and target points for intervention. Reading and Writing. 2012; 25(8):1929–1945. https://doi.org/10.1007/s11145-011-9307-x
19. Booth J, Tomporowski P, Boyle J, Ness AR, Joinson C, Leary SD, et al. Obesity impairs academic attainment in adolescence: Findings from ALSPAC, a UK cohort. International Journal of Obesity. 2014; 38(10):1335–1342. https://doi.org/10.1038/ijo.2014.40 PMID: 24614099
20. Hughes A, Wade KH, Dickson M, Rice F, Davies A, Davies NM, et al. Common health conditions in childhood and adolescence, school absence, and educational attainment: Mendelian randomization study. npj Science of Learning. 2021; 6(1):1–9. https://doi.org/10.1038/s41539-020-00080-6 PMID: 33398003
21. Nennstiel R. No Matthew effects and stable SES gaps in math and language achievement growth throughout schooling: Evidence from Germany. European Sociological Review. 2023; 39:724–740. https://doi.org/10.1093/esr/jcac062
22. Schleicher A. PISA 2018. Insights and interpretations. OECD Publishing; 2018. Available from: https://learningportal.iiep.unesco.org/en/library/pisa-2018-insights-and-interpretations.
23. Baye A, Monseur C. Gender differences in variability and extreme scores in an international context. Large-scale Assessments in Education. 2016; 4(1):1. https://doi.org/10.1186/s40536-015-0015-x
24. Darley JM, Gross PH. A hypothesis-confirming bias in labeling effects. Journal of Personality and Social Psychology. 1983; 44(1):20–33. https://doi.org/10.1037/0022-3514.44.1.20
25. Ready DD, Wright DL. Accuracy and Inaccuracy in Teachers' Perceptions of Young Children's Cognitive Abilities: The Role of Child Background and Classroom Context. American Educational Research Journal. 2011; 48(2):335–360. https://doi.org/10.3102/0002831210374874
26. Hanna RN, Linden LL. Discrimination in Grading. American Economic Journal: Economic Policy. 2012; 4(4):146–68. https://doi.org/10.1257/pol.4.4.146

27. Becker R, Jäpel F, Beck M. Diskriminierung durch Lehrpersonen oder herkunftsbedingte Nachteile von Migranten im Deutschschweizer Schulsystem? Swiss Journal of Sociology. 2013; 39(3):517–549. Available from: https://boris.unibe.ch/44457/.

28. Alesina A, Carlana M, La Ferrara E, Pinotti P. Revealing Stereotypes: Evidence from Immigrants in Schools. National Bureau of Economic Research; 2018. 25333. Available from: http://www.nber.org/papers/w25333.

29. Helbig M, Morar T. Why Teachers Assign Socially Unequal Marks. A Case for Establishing Tertiary Origin Effects in the Model of Primary and Secondary Effects of Educational Research. International Journal of Learning, Teaching and Educational Research. 2018; 17(7). https://doi.org/10.26803/ijlter.17.7.1

30. Gortazar L, Martinez de Lafuente D, Vega-Bayo A. Comparing teacher and external assessments: Are boys, immigrants, and poorer students undergraded? Teaching and Teacher Education. 2022; 115:103725. https://doi.org/10.1016/j.tate.2022.103725

31. Gil-Hernandez C, Pañeda-Fernández I, Salazar L, Castaño Muñoz J. Teacher Bias in Assessments by Student Ascribed Status: A Factorial Experiment on Discrimination and Cultural Reproduction, European Commission, JRC136851; 2024. Available from: https://publications.jrc.ec.europa.eu/repository/handle/JRC136851.

32. Burgess S, Greaves E. Test Scores, Subjective Assessment, and Stereotyping of Ethnic Minorities. Journal of Labor Economics. 2013; 31(3):535–576. https://doi.org/10.1086/669340

33. Sprietsma M. Discrimination in grading: Experimental evidence from primary school teachers. Empirical Economics. 2013; 45(1):523–538. https://doi.org/10.1007/s00181-012-0609-x

34. Botelho F, Madeira RA, Rangel MA. Racial Discrimination in Grading: Evidence from Brazil. American Economic Journal: Applied Economics. 2015; 7(4):37–52. https://doi.org/10.1257/app.20140352

35. Meissel K, Meyer F, Yao ES, Rubie-Davies CM. Subjectivity of teacher judgments: Exploring student characteristics that influence teacher judgments of student ability. Teaching and Teacher Education. 2017; 65:48–60. https://doi.org/10.1016/j.tate.2017.02.021

36. Kleen H, Bonefeld M, Glock S, Dickhäuser O. Implicit and explicit attitudes toward Turkish students in Germany as a function of teachers' ethnicity. Social Psychology of Education. 2019; 22(4):883–899. https://doi.org/10.1007/s11218-019-09502-9

37. Bonefeld M, Dickhäuser O. (Biased) grading of students' performance: Students' names, performance level, and implicit attitudes. Frontiers in Psychology. 2018; 9:481. https://doi.org/10.3389/fpsyg.2018.00481 PMID: 29867618

38. Quinn DM. Experimental evidence on teachers' racial bias in student evaluation: The role of grading scales. Educational Evaluation and Policy Analysis. 2020; 42(3):375–392. https://doi.org/10.3102/0162373720932188

39. Karnehed N, Rasmussen F, Hemmingsson T, Tynelius P. Obesity and attained education: Cohort study of more than 700,000 Swedish men. Obesity. 2006; 14:1421–1428. https://doi.org/10.1038/oby.2006.161 PMID: 16988085

40. MacCann C, Roberts RD. Just as smart but not as successful: Obese students obtain lower school grades but equivalent test scores to nonobese students. International Journal of Obesity. 2013; 37(1):40–46. https://doi.org/10.1038/ijo.2012.47 PMID: 22531092

41. Zavodny M. Does weight affect children's test scores and teacher assessments differently? Economics of Education Review. 2013; 34:135–145. https://doi.org/10.1016/j.econedurev.2013.02.003 PMID: 24014932

42. Finn KE, Seymour CM, Phillips AE. Weight bias and grading among middle and high school teachers. British Journal of Educational Psychology. 2020; 90(3):635–647. https://doi.org/10.1111/bjep.12322 PMID: 31654405

43. Lavy V. Do gender stereotypes reduce girls' or boys' human capital outcomes? Evidence from a natural experiment. Journal of Public Economics. 2008; 92(10):2083–2105. https://doi.org/10.1016/j.jpubeco.2008.02.009

44. Hofer SI. Studying gender bias in physics grading: The role of teaching experience and country. International Journal of Science Education. 2015; 37(17):2879–2905. https://doi.org/10.1080/09500693.2015.1114190

45. Di Pasquale R, Celsi L. Stigmatization of overweight and obese peers among children. Frontiers in Psychology. 2017; 8:524. https://doi.org/10.3389/fpsyg.2017.00524 PMID: 28473781

46. Lavy V, Megalokonomou R. Persistency in teachers' grading bias and effects on longer-term outcomes: University admissions exams and choice of field of study. National Bureau of Economic Research; 2019. Available from: https://doi.org/10.1016/j.econedurev.2013.02.003.

47. Angelo C, Reis AB. Gender gaps in different grading systems. Education Economics. 2020; 29(1):105–119. https://doi.org/10.1080/09645292.2020.1853681

48. Avitzour E, Choen A, Joel D, Lavy V. On the Origins of Gender-Biased Behavior: The Role of Explicit and Implicit Stereotypes. National Bureau of Economic Research; 2020. Available from: https://www.nber.org/papers/w27818.

49. Di Liberto A, Casula L, Pau S. Grading practices, gender bias and educational outcomes: Evidence from Italy. Education Economics. 2022; 30(5):481–508. https://doi.org/10.1080/09645292.2021.2004999

50. Lievore I, Triventi M. Do teacher and classroom characteristics affect the way in which girls and boys are graded? British Journal of Sociology of Education. 2023; 44(1):97–122. https://doi.org/10.1080/01425692.2022.2122942

51. Marcenaro-Gutierrez OD, Prieto-Latorre C, Rodriguez MIS. Gender differences between teachers' assessments and test based assessments. Evidence from Spain. Assessment in Education: Principles, Policy & Practice. 2023; 30(3-4):320–345. https://doi.org/10.1080/0969594X.2023.2251715

52. Schuessler J, Sønderskov KM. Compensating Discrimination: Behavioral Evidence from Danish School Registers. SocArXiv. 2023; Available from: https://doi.org/10.31235/osf.io/5zm87.

53. Van Ewijk R. Same work, lower grade? Student ethnicity and teachers' subjective assessments. Economics of Education Review. 2011; 30(5):1045–1058. https://doi.org/10.1016/j.econedurev.2011.05.008

54. Ferman B, Fontes LF. Assessing Knowledge or Classroom Behavior? Evidence of Teachers' Grading Bias. Available at SSRN 3797725. 2021;. https://doi.org/10.2139/ssrn.3797725

55. Sabia JJ, Rees DI. Body weight, mental health capital, and academic achievement. Review of Economics of the Household. 2015; 13(3):653–684. https://doi.org/10.1007/s11150-014-9272-7

56. Crenshaw K. Demarginalizing the Intersection of Race and Sex: A Black Feminist Critique of Antidiscrimination Doctrine, Feminist Theory and Antiracist Politics. University of Chicago Legal Forum. 1989;1989(1). Available from: https://chicagounbound.uchicago.edu/uclf/vol1989/iss1/8/.

57. Gottburgsen A, Gross C. Welchen Beitrag leistet "Intersektionalität" zur Klärung von Kompetenzunterschieden bei Jugendlichen? In: Becker R, Solga H, editors. Soziologische Bildungsforschung. Wiesbaden: Springer Fachmedien Wiesbaden; 2012. p. 86–110. https://doi.org/10.1007/978-3-658-00120-9_4

58. Bécares L, Priest N. Understanding the Influence of Race/Ethnicity, Gender, and Class on Inequalities in Academic and Non-Academic Outcomes among Eighth-Grade Students: Findings from an Intersectionality Approach. PLOS ONE. 2015; 10(10):e0141363. https://doi.org/10.1371/journal.pone.0141363 PMID: 26505623

59. Gross C, Gottburgsen A, Phoenix A. Education systems and intersectionality. In: Education Systems and Inequalities: International Comparisons. Policy Press; 2016. p. 51–72. https://doi.org/10.1332/policypress/9781447326106.003.0004

60. Nichols S, Stahl G. Intersectionality in higher education research: A systematic literature review. Higher Education Research & Development. 2019; 38(6):1255–1268. https://doi.org/10.1080/07294360.2019.1638348

61. McPherson K. Black girls are not magic; they are human: Intersectionality and inequity in the Greater Toronto Area (GTA) schools. Curriculum Inquiry. 2020; 50(2):149–167. https://doi.org/10.1080/03626784.2020.1729660

62. Kunda Z, Spencer SJ. When do stereotypes come to mind and when do they color judgment? A goal-based theoretical framework for stereotype activation and application. Psychological Bulletin. 2003; 129 (4):522–544. https://doi.org/10.1037/0033-2909.129.4.522 PMID: 12848219

63. Dardanoni V, Modica S, Pennsi A. Grading Across Schools. The BE Journal of Economic Analysis & Policy. 2009; 9(1). https://doi.org/10.2202/1935-1682.2065

64. Hinnerich BT, Höglin E, Johannesson M. Are boys discriminated in Swedish high schools? Economics of Education Review. 2011; 30(4):682–690. https://doi.org/10.1016/j.econedurev.2011.02.007

65. Wenz SE, Hoenig K. Ethnic and social class discrimination in education: Experimental evidence from Germany. Research in Social Stratification and Mobility. 2020; 65:100461. https://doi.org/10.1016/j.rssm.2019.100461

66. Matthews JS, Ponitz CC, Morrison FJ. Early gender differences in self-regulation and academic achievement. Journal of Educational Psychology. 2009; 101(3):689. https://doi.org/10.1037/a0014240

67. Duckworth AL, Seligman ME. Self-discipline gives girls the edge: Gender in self-discipline, grades, and achievement test scores. Journal of Educational Psychology. 2006; 98(1):198. https://doi.org/10.1037/0022-0663.98.1.198

68. Gilgen S, Stocker M. Discrimination at the Crossroads? Evidence from a Factorial Survey Experiment on Teacher's Tracking Decisions. Swiss Journal of Sociology. 2022; 48(1):77–105. https://doi.org/10.2478/sjs-2022-0005

69. Glock S, Böhmer I. Teachers' and preservice teachers' stereotypes, attitudes, and spontaneous judgments of male ethnic minority students. Studies in Educational Evaluation. 2018; 59:244–255. https://doi.org/10.1016/j.stueduc.2018.09.001

70. McKown C, Weinstein RS. Teacher expectations, classroom context, and the achievement gap. Journal of School Psychology. 2008; 46(3):235–261. https://doi.org/10.1016/j.jsp.2007.05.001 PMID: 19083359

71. Glock S. Does ethnicity matter? The impact of stereotypical expectations on in-service teachers' judgments of students. Social Psychology of Education. 2016; 19(3):493–509. https://doi.org/10.1007/s11218-016-9349-7

72. Schneider T. Die Bedeutung der sozialen Herkunft und des Migrationshintergrundes für Lehrerurteile am Beispiel der Grundschulempfehlung. Zeitschrift für Erziehungswissenschaft. 2011; 14:371–396. https://doi.org/10.1007/s11618-011-0221-4

73. Batruch A, Geven S, Kessenich E, Van De Werfhorst HG. Are tracking recommendations biased? A review of teachers' role in the creation of inequalities in tracking decisions. Teaching and Teacher Education. 2023; 123:103985. https://doi.org/10.1016/j.tate.2022.103985

74. Landy D, Sigall H. Beauty is talent: Task evaluation as a function of the performer's physical attractiveness. Journal of Personality and Social Psychology. 1974; 29(3):299. https://doi.org/10.1037/h0036018

75. Yu B. Kindergarten Obesity and Academic Achievement: The Mediating Role of Weight Bias. Frontiers in Psychology. 2021; 12:1147. https://doi.org/10.3389/fpsyg.2021.640474 PMID: 33935891

76. Budowski M, Hadjar A. Intersectionality and Social Inequalities Beyond Social Origin. Socialpolicych. 2023; 1(1). Available from: https://www.sozialpolitik.ch/article/view/4018/3659.

77. Crenshaw K. Mapping the Margins: Intersectionality, Identity Politics, and Violence against Women of Color. Stanford Law Review. 1991; 43(6):1241. https://doi.org/10.2307/1229039

78. Munsch C, Gemende M, Rotino SWU, editors. Eva ist emanzipiert, Mehmet ist ein Macho: Zuschreibung, Ausgrenzung, Lebensbewältigung und Handlungsansätze im Kontext von Migration und Geschlecht. Geschlechterforschung. Juventa; 2007.

79. Heiserman N. Complex Stereotypes: Stereotypes Across the Intersections of Gender, Sexuality, Age, Race/Ethnicity, and Social Class [PhD thesis]. University of South Carolina; 2023. Available from: https://scholarcommons.sc.edu/cgi/viewcontent.cgi?article=8117&context=etd.

80. Ghavami N, Peplau LA. An Intersectional Analysis of Gender and Ethnic Stereotypes: Testing Three Hypotheses. Psychology of Women Quarterly. 2012; 37(1):113–127. https://doi.org/10.1177/0361684312464203

81. Steinhauer HW, Zinn S. NEPS technical report for weighting: Weighting the sample of starting cohort 4 of the National Educational Panel Study (wave 1 to 6). NEPS Survey Paper Number 2. 2016;. https://doi.org/10.5157/NEPS:SP02:1.0

82. NEPS Network. National Educational Panel Study, Scientific Use File of Starting Cohort Grade 9. Leibniz Institute for Educational Trajectories (LIfBi), Bamberg. 2023. https://doi.org/10.5157/NEPS:SC4:13.0.0

83. Blossfeld HP, Roßbach HG, editors. Education as a lifelong process: The German National Educational Panel Study (NEPS). Edition ZfE (2nd ed.). Springer VS.; 2019. https://doi.org/10.1007/978-3-658-23162-0

84. Sticca F, Goetz T, Bieg M, Hall NC, Eberle F, Haag L. Examining the accuracy of students' self-reported academic grades from a correlational and a discrepancy perspective: Evidence from a longitudinal study. PLOS ONE. 2017; 12(11):e0187367. https://doi.org/10.1371/journal.pone.0187367 PMID: 29112979

85. Kuncel NR, Credé M, Thomas LL. The Validity of Self-Reported Grade Point Averages, Class Ranks, and Test Scores: A Meta-Analysis and Review of the Literature. Review of Educational Research. 2005; 75(1):63–82. https://doi.org/10.3102/00346543075001063

86. Weinert S, Artelt C, Prenzel M, Senkbeil M, Ehmke T, Carstensen CH, et al. Development of Competencies Across the Life Course. In: Blossfeld HP, Roßbach HG, editors. Education as a Lifelong Process. The German National Educational Panel Study (NEPS). Springer Fachmedien Wiesbaden; 2019. p. 57–81. https://doi.org/10.1007/978-3-658-23162-0_4

87. CDC—National Center for Health Statistics. BMI-for-age charts, 2 to 20 years, LMS parameters and selected smoothed BMI (kilograms/meters squared) percentiles, by sex and age. 2000. Available from: https://www.cdc.gov/growthcharts/data/zscore/bmiagerev.csv.

88. Ganzeboom HB. A new International Socio-Economic Index (ISEI) of Occupational Status for the International Standard Classification of Occupation 2008 (ISCO-08) constructed with data from the ISSP 2002-2007. In: Annual Conference of International Social Survey Programme, Lisbon. vol. 1; 2010.

89. Goodman R. The Strengths and Difficulties Questionnaire: A research note. Journal of Child Psychology and Psychiatry. 1997; 38(5):581–586. https://doi.org/10.1111/j.1469-7610.1997.tb01545.x PMID: 9255702

**90.** Morgan JF, Reid F, Lacey JH. The SCOFF questionnaire: Assessment of a new screening tool for eating disorders. BMJ. 1999; 319(7223):1467–1468. https://doi.org/10.1136/bmj.319.7223.1467 PMID: 10582927

**91.** Nennstiel R. Positive Ethnic Choice Effects are Persistent Over Time but not Across Minority Generations: Evidence from Switzerland. KZfSS Kölner Zeitschrift für Soziologie und Sozialpsychologie. 2022; 74(4):499–524. https://doi.org/10.1007/s11577-022-00865-0

**92.** Steele CM, Aronson J. Stereotype threat and the intellectual test performance of African Americans. Journal of Personality and Social Psychology. 1995; 69(5):797–811. https://doi.org/10.1037/0022-3514.69.5.797 PMID: 7473032

**93.** Appel M, Weber S, Kronberger N. The influence of stereotype threat on immigrants: Review and meta-analysis. Frontiers in Psychology. 2015; 6:900. https://doi.org/10.3389/fpsyg.2015.00900 PMID: 26217256

**94.** Froehlich L, Mok SY, Martiny SE, Deaux K. Stereotype threat-effects for Turkish-origin migrants in Germany: Taking stock of cumulative research evidence. European Educational Research Journal. 2018; p. 1–25. https://doi.org/10.1177/1474904118807539
